# Adaptive evolution of sesquiterpene deoxyphomenone in mycoparasitism by *Hansfordia pulvinata* associated with horizontal gene transfer from *Aspergillus* species

**DOI:** 10.1128/mbio.04007-24

**Published:** 2025-03-20

**Authors:** Kazuya Maeda, Takuya Sumita, Oumi Nishi, Hirotoshi Sushida, Yumiko Higashi, Hiroyuki Nakagawa, Tomoko Suzuki, Eishin Iwao, Much Zaenal Fanani, Yoshiaki Nishiya, Yuichiro Iida

**Affiliations:** 1 Laboratory of Plant Pathology, Setsunan University, Hirakata, Osaka, Japan; 2 Laboratory of Environmental Microbiology, Setsunan University 47731 , Neyagawa, Osaka, Japan; 3 National Agriculture and Food Research Organization (NARO), Tsu, Mie, Japan; 4 Institute of Food Research, National Agriculture and Food Research Organization (NARO), Tsukuba, Ibaraki, Japan; 5 Research Center for Advanced Analysis, Core Technology Research Headquarters, National Agriculture and Food Research Organization (NARO), Tsukuba, Ibaraki, Japan; 6 Department of Chemical Biological Sciences, Japan Women’s University, Bunkyo-ku, Tokyo, Japan; Vallabhbhai Patel Chest Institute, Delhi, India

**Keywords:** mycoparasite, deoxyphomenone, sporogen-AO 1, horizontal gene transfer, *Aspergillus flavus*, *Cladosporium fulvum*, *Hansfordia pulvinata*

## Abstract

Leaf mold caused by the ascomycete fungus *Cladosporium fulvum* is a devastating disease of tomato plants. The mycoparasitic fungus *Hansfordia pulvinata* is an effective biocontrol agent that parasitizes *C. fulvum* hyphae on leaves and secretes 13-deoxyphomenone, an eremophilane-type sesquiterpene, which was also identified as a sporulation-inducing factor in *Aspergillus oryzae*. Here, we identified deoxyphomenone biosynthesis (*DPH*) gene clusters conserved in both *H. pulvinata* and *Aspergillus* section *Flavi*, including *A. oryzae* and *A. flavus*. Functional disruption of *DPH1* orthologous genes encoding sesquiterpene cyclase in *H. pulvinata*, *A. oryzae*, and its close relative *A. flavus* revealed that deoxyphomenone in *H. pulvinata* had exogenic antifungal activity against *C. fulvum* and controlled endogenic sporulation in *Aspergillus* species. Complete *DPH* clusters, highly similar to those in *H. pulvinata*, were exclusive to *Aspergillus* section *Flavi*, while species in other *Aspergillus* sections contained fragmented *DPH* clusters. A comparative genomics analysis revealed that these *DPH* gene clusters share a common origin and are horizontally transferred from an ancestor of *Aspergillus* to *H. pulvinata*. Our results suggest that after horizontal transfer, *H. pulvinata* maintained the *DPH* cluster as the inhibitory effect of deoxyphomenone on spore germination and mycelial growth contributed to its mycoparasitism on the host fungus *C. fulvum*.

**IMPORTANCE:**

Tomato leaf mold disease caused by *C. fulvum* poses a significant economic threat to tomato production globally. Breeders have developed tomato cultivars with *Cf* resistance genes. *C. fulvum* frequently evolves new races that overcome these genetic defenses, complicating control efforts. Additionally, the pathogen has developed resistance to chemical fungicides, prompting the need for sustainable alternatives like biocontrol agents. The mycoparasitic fungus *H. pulvinata* is crucial as an effective agent against *C. fulvum*. Clarifying the mechanism of mycoparasitism is significant, as it enhances its application as a biocontrol agent against plant pathogens. This study revealed how *H. pulvinata* produces deoxyphomenone, an antifungal compound, through horizontal gene transfer from *Aspergillus* species. It is hypothesized that mycoparasitism could be one of the mechanisms that facilitated horizontal gene transfer between fungi. These insights facilitate the development of eco-friendly, sustainable agricultural practices by reducing dependence on chemical fungicides and promoting natural pathogen control methods.

## INTRODUCTION

The biotrophic filamentous fungus *Cladosporium fulvum* (Cooke) (syn. *Passalora fulva*, *Fulvia fulva*), the causal agent of leaf mold of tomato, is an economic problem in most countries that grow tomatoes (1, 2). Colonization of tomato leaves by *C. fulvum* is facilitated by numerous effector proteins that are secreted into the apoplast by the fungus (3). To combat *C. fulvum*, breeders have introduced *Cf* resistance genes, which encode receptor-like proteins with extracytoplasmic leucine-rich repeats, into commercial tomato cultivars from wild relatives (2, 3). Tomato *Cf* gene-encoded receptor-like proteins recognize corresponding effector proteins of *C. fulvum* as virulence factors in the apoplastic region and trigger a hypersensitive response that rapidly kills plant cells near the infection site, inhibiting the growth of the biotrophic pathogen and preventing its spread (4). However, the occasional loss or mutation of effector genes allows *C. fulvum* to escape the host immune system, resulting in the emergence of new races (5, 6). Although single resistance genes have been introduced into many tomato cultivars, overuse of resistant cultivars with only one or a few *Cf* genes has led to the development of *C. fulvum* races that have overcome these resistance genes. A further problem is that *C. fulvum* has developed resistance to several fungicides (7, 8). Thus, the use of antagonistic microorganisms instead of synthetic fungicides is desired for sustainable control of leaf mold.

**Fig 1:**
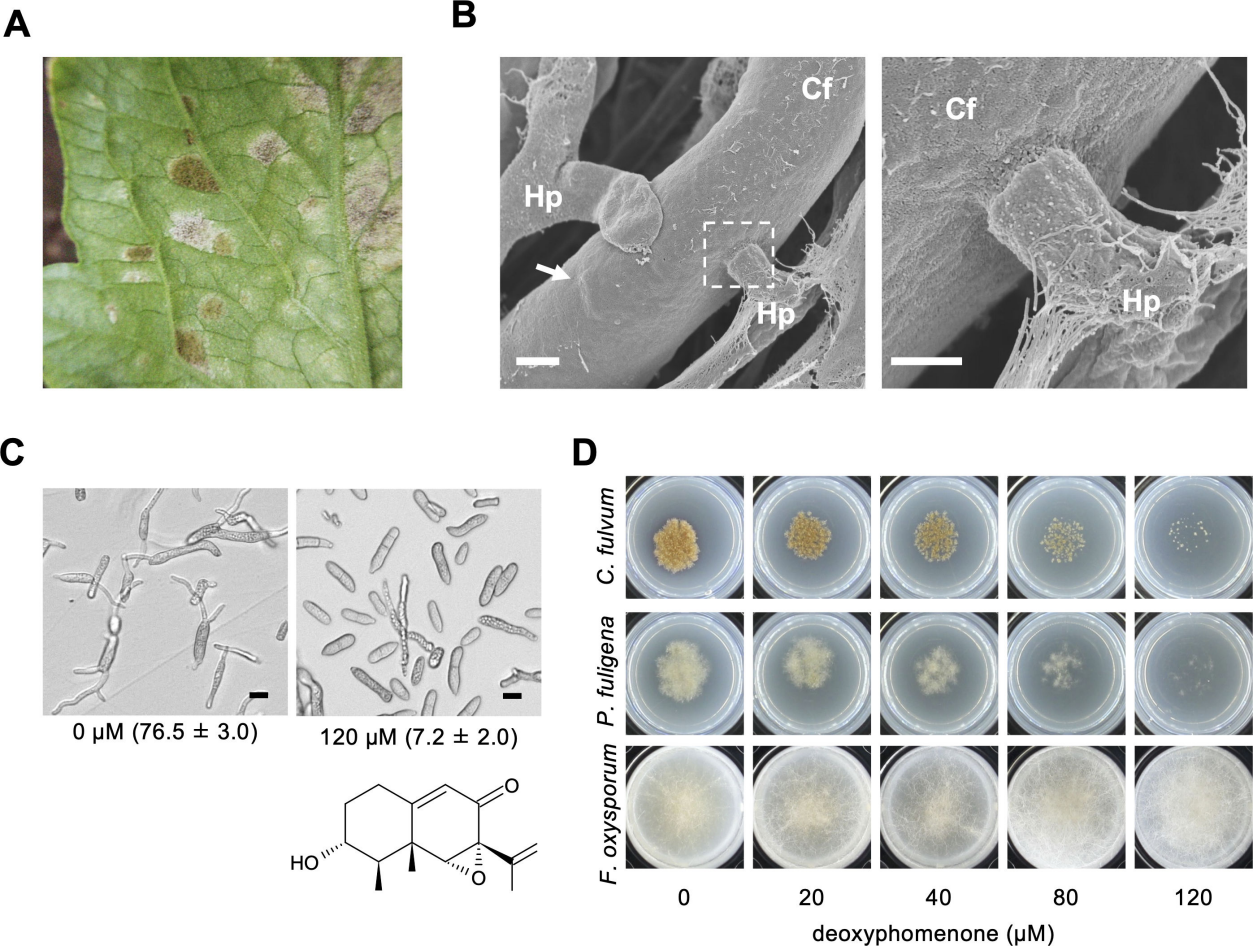
Deoxyphomenone produced by the mycoparasite *Hansfordia pulvinata* is an antifungal sesquiterpene active against the tomato leaf mold pathogen, *Cladosporium fulvum*. (**A**) Leaf mold symptoms (brown lesions) caused by *C. fulvum* and lesions covered by white mycelia of *H. pulvinata* on a tomato leaf. (**B**) Scanning electron micrographs of the hyphae of *H. pulvinata* parasitizing hyphae of *C. fulvum*. Right: detail of area in dotted box in left image. Arrow: bulge in hypha of *C. fulvum* caused by hyphae of *H. pulvinata*. (**C**) Germinated spores of *C. fulvum* 24 h after treatment with 120 µM deoxyphomenone or with 1% methanol (vol/vol) as a control (0 µM deoxyphomenone). Germination percentages are in parentheses. The chemical structure of deoxyphomenone is also shown. (**D**) Antifungal activities of various concentrations of deoxyphomenone in MM agar on mycelial growth of tomato pathogenic fungi (*C. fulvum*, *Pseudocercospora fuligena*, and *Fusarium oxysporum* f. sp. *lycopersici*). Bars = 10 µm.

We serendipitously discovered white mycelia growing over lesions of leaf mold-infected tomato leaves in a greenhouse and found that disease progression was inhibited on these leaves (Fig. 1A) (9). In further studies, we showed that the fungus parasitizes hyphae of *C. fulvum* and inhibited all known races of this fungus. The mycoparasitic fungus was identified as *Hansfordia pulvinata* (Berk. & M.A. Curtis) S. Hughes [syn. *Dicyma pulvinata*] (10–13) based on its morphological characteristics and a phylogenetic analysis (9). *H. pulvinata* produces 13-deoxyphomenone, an eremophilane-type sesquiterpene, as an antifungal compound during vegetative growth (14). Around the same time, this compound was also found and characterized (but named sporogen-AO 1) from *Aspergillus oryzae*, which is used to produce koji that is used for the production of many Japanese fermented foods, such as Japanese sake and soy sauce (15, 16). This sesquiterpene is toxic to *C. fulvum*, which is parasitized by *H. pulvinata,* but it induces spore production in *A. oryzae*. However, its biosynthetic pathway in these fungi, the molecular basis of its antifungal activity and spectrum, and the specificity of its sporogenic activity in different *Aspergillus* species have not been elucidated.

Because deoxyphomenone is produced by most strains of *H. pulvinata* and might be widely produced among *Aspergillus* species, it may have mycoparasitic and/or ecological functions in these fungi. In this study, we investigated the roles of deoxyphomenone in *H. pulvinata* and *Aspergillus* species. The antifungal spectrum of the compound was determined for *C. fulvum* and other tomato fungal pathogens that are not host fungi of *H. pulvinata*, such as *Pseudocercospora fuligena*, the causal agent of tomato black leaf mold, which is phylogenetically closely related to *C. fulvum*, and the soilborne tomato pathogen *Fusarium oxysporum* f. sp. *lycopersici*. We identified deoxyphomenone biosynthetic gene clusters in *H. pulvinata* and *Aspergillus* genome sequences and functionally disrupted sesquiterpene cyclase genes to reveal the main function of deoxyphomenone. Furthermore, we used a comparative genomics approach to elucidate the origin of these gene clusters in *Aspergillus* and *H. pulvinata*.

## RESULTS

### Antifungal activity of deoxyphomenone against plant pathogens

*H. pulvinata* parasitizes *C. fulvum* hyphae in liquid culture without a carbon source (9). In present tests, *H. pulvinata* parasitized *C. fulvum* when grown on nylon membranes on nutrient-poor water agar. Scanning electron micrographs showed that hyphae of strain 414-3 penetrated the cell wall of *C. fulvum* (Fig. 1B).

Since strain 414-3 produces about 100 µM deoxyphomenone in minimal medium (MM) broth (9), we next evaluated its antifungal activity against *C. fulvum* using 120 µM commercial deoxyphomenone. At 120 µM, the compound inhibited colony formation and hyphal elongation (Fig. S1A and B). Deoxyphomenone also strongly inhibited spore germination at 120 µM, which was only 7.2% after 24 h compared with 76.5% for the control spores treated with 1% (vol/vol) methanol (Fig. 1C). When *C. fulvum* spores were treated with the fungicide captan at 100 µM, and after 24 h captan was replaced by distilled water, almost all failed to germinate, but when deoxyphomenone (120 µM) was replaced by distilled water 24 h after treatment, about half of the treated spores germinated (Fig. S1C). Thus, the activity of deoxyphomenone is fungistatic; it inhibits the germination of *C. fulvum* spores but does not kill them. Deoxyphomenone treatment did not affect the structure of intracellular organelles or the cell membrane of the spores or induce necrosis in tomato leaves, even at concentrations as high as 80 µM, contrary to a previous report (14) (Fig. S1D and E). When plated on MM agar, external application of deoxyphomenone also inhibited colony formation of plant pathogenic fungi that are not hosts of *H. pulvinata*: the growth of *P. fuligena* phylogenetically closely related to *C. fulvum* was inhibited at the same concentrations as used for *C. fulvum*, but growth of *F. oxysporum* f. sp. *lycopersici* was not affected (Fig. 1D).

### Deoxyphomenone biosynthetic gene clusters are conserved between *H. pulvinata* and *Aspergillus*

We searched for homologous genes in the genome sequences of *H. pulvinata* 414-3 and *A. oryzae* RIB40 (Table 1) using the amino acid sequence of aristolocene synthase Ari1 from *Penicillium roqueforti* (17) since the structure of deoxyphomenone has an aristolochene-like backbone (Fig. 1C) and found two candidates (gene IDs, DIP_001954 and DIP_006271) in *H. pulvinata* (18) and one (GenBank accession XP_023093357) in *A. oryzae*. Phylogenetic comparisons with other fungal sesquiterpene cyclases placed DIP_001954 and XP_023093357 in the same clade as enzymes that catalyze the 1,10-cyclization of farnesyl diphosphate, a common sesquiterpene precursor (Fig. S2). The deduced amino acid sequences of DIP_001954 and XP_023093357 had 61.6% similarity (49.1% identity) (Fig. S3). We named DIP_001954 “*HpDPH1*” (deoxyphomenone biosynthesis gene 1) and the homologous gene XP_023093357 “*AoDPH1*”.

**TABLE 1:** Strains used in this study

Strain name	Description	Plasmid^*a*^
*Hasfordia pulvinata*		
414-3	Wild type	
KO10	Mutation in the *HpDPH1* gene	pPM43GW (Pgpd1::*NPTII*, Ptoxa::GFP)
KO37	Mutation in the *HpDPH1* gene	pPM43GW (Pgpd1::*NPTII*, Ptoxa::GFP)
EC1	Ectopic transformant	pPM43GW (Pgpd1::*NPTII*, Ptoxa::GFP)
EC6	Ectopic transformant	pPM43GW (Pgpd1::*NPTII*, Ptoxa::GFP)
CO10A	KO10 complemented with the *AoDPH1* gene	pPZP (Ptrpc::*AoDPH1*, Pgpd1::*HPH*)
*Aspergillus oryzae*		
RIB40	Wild type	
4-17D	Mutation in the *AoDPH1* gene	pSH75 (Pgpda::*PtrA*)
3-3E	Ectopic transformant	pSH75 (Pgpda::*PtrA*)
4-1E	Ectopic transformant	pSH75 (Pgpda::*PtrA*)
*Aspergillus flavus*		
NBRC 114564	Wild type	
Aft12	Mutation in the *AfDPH1* gene	pSH75 (Pgpda::*PtrA*)
Aft27	Mutation in the *AfDPH1* gene	pSH75 (Pgpda::*PtrA*)
Aft32	Mutation in the *AfDPH1* gene	pSH75 (Pgpda::*PtrA*)
Aft5	Ectopic transformant	pSH75 (Pgpda::*PtrA*)
Aft26	Ectopic transformant	pSH75 (Pgpda::*PtrA*)

^*a*^ Pgpd1, *Cochliobolus heterostrophus gpd1* promoter; *NPTII*, G418 resistance gene; Ptoxa, *Pyrenophora tritici-repentis toxA* promoter; *HPH,* hygoromycin resistance gene; Pgpda, *Aureobasidium nidulans gpdA* promoter; *PtrA*, pyrithiamine resistance gene.

**Fig 2:**
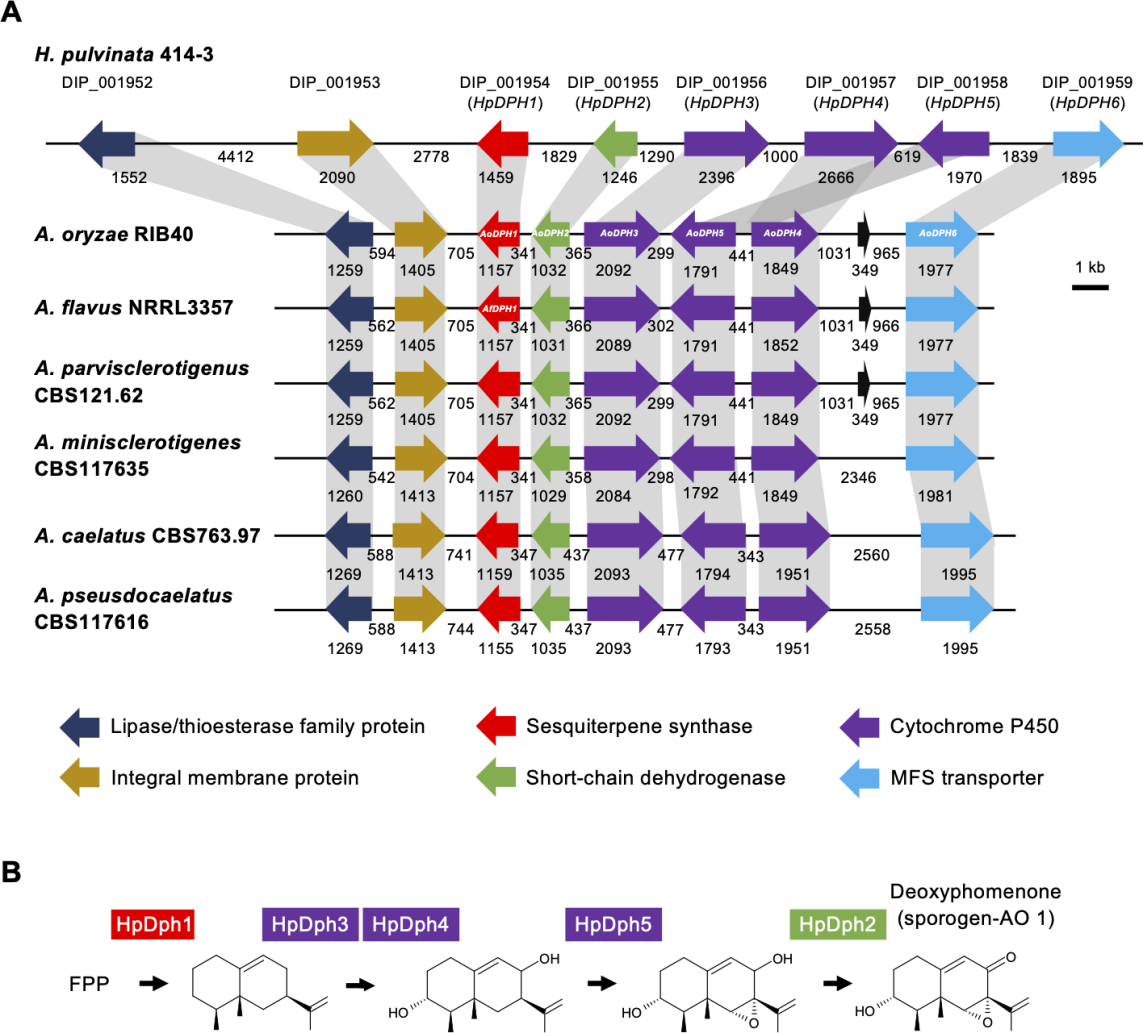
Synteny of the deoxyphomenone biosynthetic (*DPH*) gene clusters conserved in the mycoparasite *Hansfordia pulvinata* and *Aspergillus* section *Flavi*. (**A**) Gene clusters conserved in *H. pulvinata* and *Aspergillus*. The original gene IDs (DIP) and names (*HpDPH1* to *HpDPH6*) of *H. pulvinata* are above the genes. Protein annotations are indicated by color. The names of the *HpDPH* homologous genes in *Aspergillus* mentioned in the text are indicated in the arrows. Numbers indicate the length of the genes and intergenic regions in base pairs. ORFs (black arrows) were detected in the upstream region of *HpDPH6* in three *Aspergillus* species, and no homologous gene sequence was found in the National Center for Biotechnology Information non-redundant database. (**B**) Predicted biosynthetic pathway of deoxyphomenone. Intermediates were logically inferred. FPP, farnesyl pyrophosphate.

In the nucleotide sequences around *HpDPH1*, we found genes encoding short-chain dehydrogenase (*HpDPH2*), three cytochrome P450s (*HpDPH3*, *HpDPH4*, *HpDPH5*), and major facilitator superfamily transporter (*HpDPH6*), altogether forming the deoxyphomenone biosynthesis (*DPH*) gene cluster. We also found homologs of these genes in *A. oryzae* (Fig. 2A). Genes encoding a lipase/thioesterase family protein and an integral membrane protein were conserved in both fungi, but their function in deoxyphomenone biosynthesis is unknown. The position and orientation of all genes were highly conserved, but the loci of *AoDPH4* and *AoDPH5* were switched (Fig. S4). We presumed that the deoxyphomenone biosynthetic pathway involved cyclization of farnesyl diphosphate by HpDph1, followed by oxygenation and epoxidation by cytochrome P450s HpDph3, HpDph4, and HpDph5 and subsequent hydroxylation by HpDph2 (Fig. 2B).

To obtain a comprehensive view of the distribution of the *DPH* cluster in other fungi, we extended the search to *HpDPH1* homologues in the MycoCosm database (19). Genes partially homologous to *HpDPH1* were found in 139 fungal genomes of the *Eurotiomycetes*, *Sordariomycetes,* and *Dothideomycetes* (Table S1). Besides the *DPH* gene cluster present in *H. pulvinata*, other *DPH* genes were detected as a gene cluster in *Aspergillus* species only (*Eurotiomycetes*) but not in the *Xylariales* (*Sordariomycetes*), to which *H. pulvinata* belongs, which suggests that the latter fungus has obtained this cluster by horizontal transfer. Notably, the cluster was completely conserved in *Aspergillus* species section *Flavi* to which *A. oryzae* belongs (Fig. S5; Fig. 2A).

**Fig 3:**
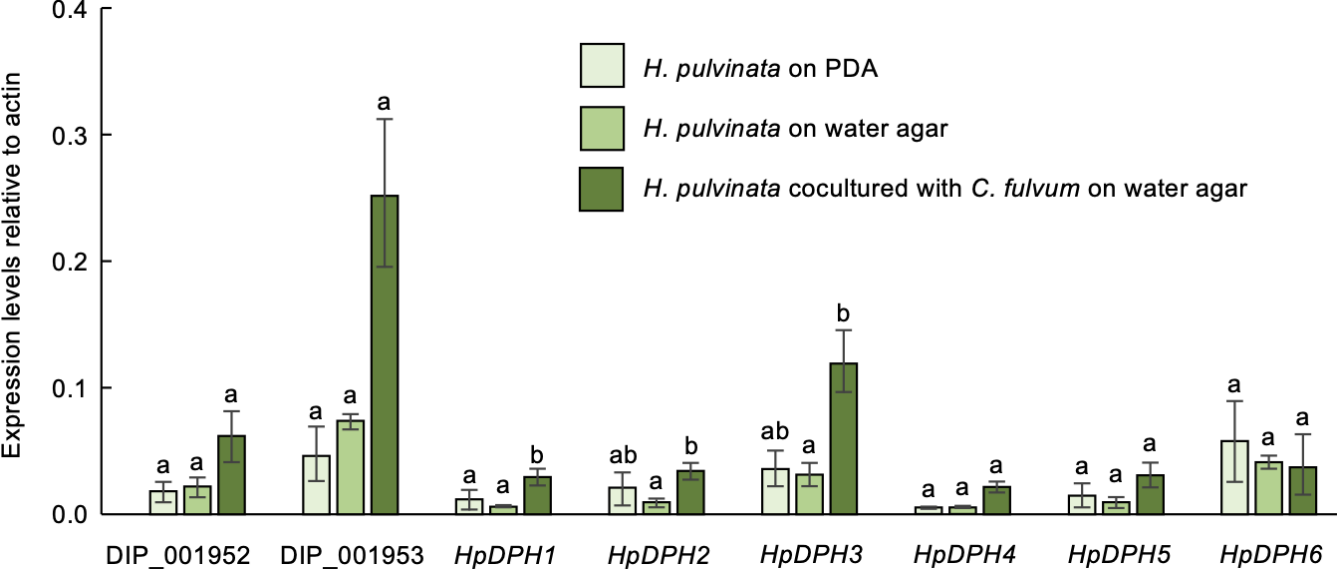
Relative gene expression levels of the deoxyphomenone biosynthesis (*DPH*) genes in the mycoparasite *Hansfordia pulvinata*. A spore suspension of *H. pulvinata* 414-3 was sprayed on *Cladosporium fulvum* on water agar (WA). *H. pulvinata* and *C. fulvum* were grown on PDA or WA as controls. Expression was quantified by qPCR and normalized using those of the *H. pulvinata* actin gene. Values are the means of three biological replicates (±standard deviation). Significant differences (*P* < 0.05) in expression for a gene among the three treatments are indicated by different letters.

We then analyzed the expression of the putative deoxyphomenone biosynthetic genes during mycoparasitism of *H. pulvinata* 414-3 *in vitro* using quantitative real-time PCR. *H. pulvinata* never parasitized *C. fulvum* on the nutrient-rich potato dextrose agar (PDA), but it did so on the nutrient-poor water agar (Fig. 2A). The expression of the *HpDPH* genes in *H. pulvinata* did not differ when grown on PDA medium or on water agar (Fig. 3). In contrast, the expression of *HpDPH* genes was significantly upregulated when *H. pulvinata* parasitized *C. fulvum* on water agar. Expressions of *HpDPH1*, *HpDPH2*, and *HpDPH3* genes, which encode sesquiterpene cyclase, short-chain dehydrogenase, and cytochrome P450, respectively, were significantly induced during mycoparasitism (Fig. 3). Expression of DIP_001952 (lipase/thioesterase family protein) and DIP_001953 (integral membrane protein) genes also increased, suggesting that these proteins are associated with deoxyphomenone biosynthesis or mycoparasitism.

### Deoxyphomenone is the main antifungal compound of *H. pulvinata* effective against *C. fulvum*

Since the *DPH* gene cluster was also found in the genus *Aspergillus*, we assayed for the presence of deoxyphomenone in the MM culture filtrate from 22 strains of *A. oryzae* and 12 strains of *A. flavus*. In contrast to *H. pulvinata* 414-3, which produced deoxyphomenone as high as about 80 µM, *A. oryzae* and *A. flavus* strains produced only low levels of deoxyphomenone: the highest concentrations in *A. oryzae* and *A. flavus* strains were about 0.8 and 3.5 µM, respectively (Fig. S6). We then measured deoxyphomenone by the most productive strains of *A. oryzae* (RIB40) and *A. flavus* (NBRC114564) in different culturing conditions and found that both strains produced the compound when grown as still cultures in MM broth (Fig. S7). Interestingly, neither strain produced deoxyphomenone at 35°C, the optimum temperature for vegetative growth and sporulation, but showed maximal production at 25°C, at which vegetative growth and sporulation are retarded. *H. pulvinata* showed the highest production of deoxyphomenone at 28°C, a temperature at which vegetative growth is also retarded (Fig. S7).

**Fig 4:**
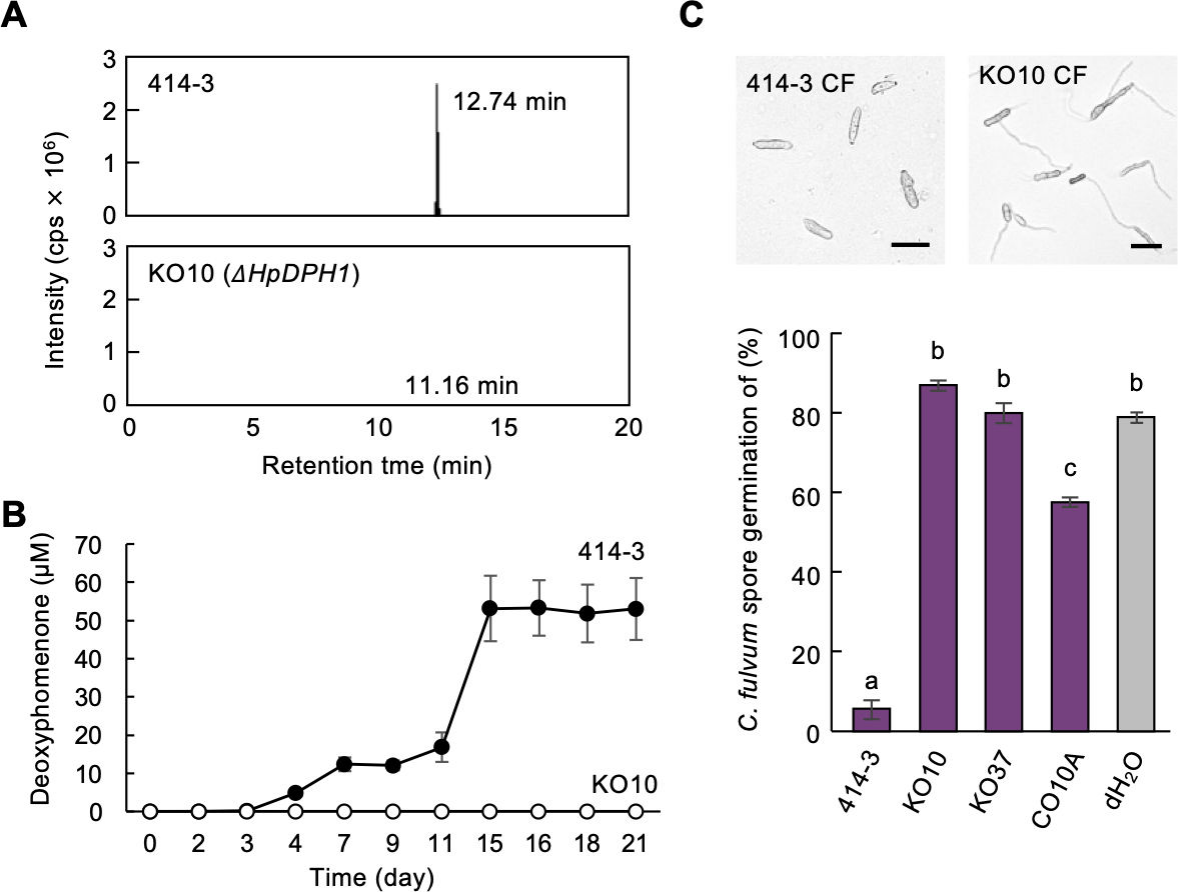
Deoxyphomenone production by wild-type and knock-out mutants of *Hansfordia pulvinata* and their antifungal activity. Wild-type 414-3 of *H. pulvinata*, *ΔHpDPH1* mutants (KO10, KO37), and *AoDPH1-*complemented KO37 (CO10A) were cultured in MM broth for 2 weeks and the filtered culture filtrate collected to (**A**) quantify deoxyphomenone production in 414-3 and mutant KO10 using LC–MS/MS. The retention time of the highest peak is given for each. (**B**) Time course of deoxyphomenone production in 414-3 and mutant KO10. (**C**) Germination of *Cladosporium fulvum* spores after 24 h of exposure to deoxyphomenone in culture filtrate (CF) of 414-3 or KO10 (bars = 20 µm). Different letters in the graph indicate significant differences (*P* < 0.05) in germination among treatments. Values are means of three replicates (±standard deviation).

We generated Δ*HpDPH1* knockout mutant strains of 414-3 by homologous recombination through *Agrobacterium tumefaciens*-mediated transformation (Fig. S8). Quantitative analysis by liquid chromatography–tandem mass spectrometry (LC–MS/MS) revealed the presence of deoxyphomenone in the culture filtrate of wild-type 414-3 strain but none in that of the *ΔHpDPH1* mutant strain KO10 (Fig. 4A and B). The culture filtrate of 14-day-old cultures of 414-3 containing deoxyphomenone strongly inhibited the germination of *C. fulvum* spores, whereas those of the Δ*HpDPH1* mutant strains KO10 and KO37 showed no inhibitory activity, suggesting that deoxyphomenone is the main compound contributing to the antifungal activity of *H. pulvinata* (Fig. 4C). The deoxyphomenone production and antifungal activity were partially restored in the loss-of-function Δ*HpDPH1* mutant strain after introduction of a functional *AoDPH1* gene (CO10A) (Fig. 4C). The Δ*HpDPH1* mutant KO10 parasitized *C. fulvum* similar to the wild-type 414-3 strain, with no differences in spore number or vegetative growth, as we expected previously (9) (Fig. S9).

### The function of deoxyphomenone differs between *H. pulvinata* and the *Aspergillus* species

**Fig 5:**
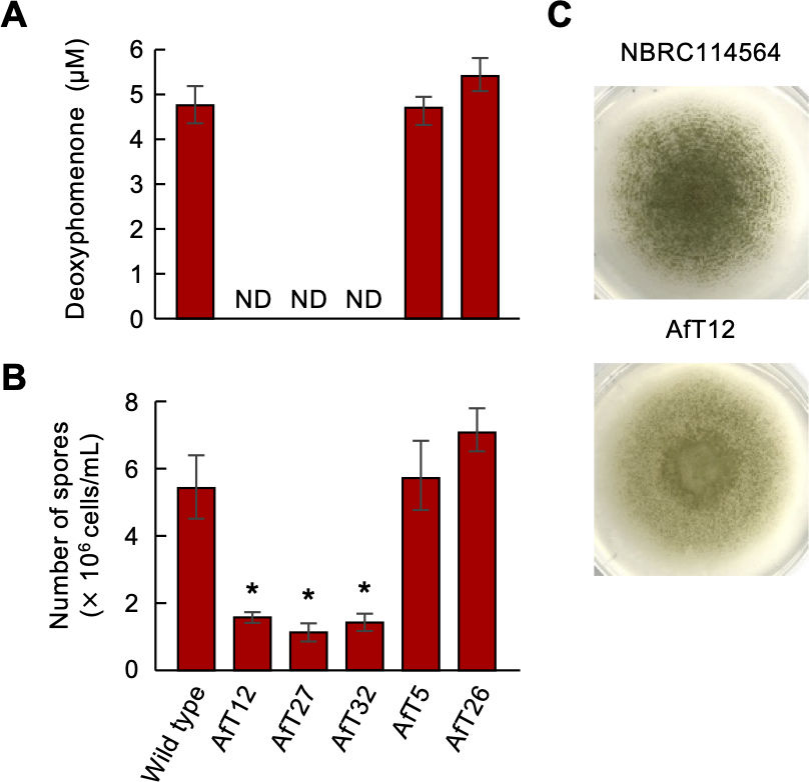
Deoxyphomenone production and sporulation by *A. flavus* wild-type NBRC114564 and Δ*AfDPH1* mutant strains and ectopic mutants. The wild type, three Δ*AfDPH1* mutants, and two ectopic mutant strains were cultured in MM broth or agar. Values are means of three replicates (±standard deviation). (**A**) LC–MS/MS analysis of deoxyphomenone in culture filtrates of the various strains. ND: not detected. (**B**) Number of spores formed on agar. Asterisk indicates significant difference (*P* < 0.05) compared to the wild type in Williams’ test. (**C**) Colony morphology of the wild type, Δ*AfDPH1* mutant, and ectopic AfT32 mutant.

We generated knockout strains of the homologous gene *AfDPH1* in *A. flavus* NBRC114564, which produced more deoxyhomenone than *A. oryze* RIB40 (Fig. S6). LC–MS/MS analysis showed that wild-type strain and ectopic transformants produced deoxyphomenone, while the Δ*AfDPH1* mutant strains produced none (Fig. 5A). Three Δ*AfDPH1* mutant strains grown on MM agar produced significantly less spores than the wild type (Fig. 5B and C). Conversely, a Δ*AoDPH1* mutant of *A. oryzae* RIB40 completely lost the ability to produce deoxyphomenone, but its spore production and colony morphology were similar to those of the wild-type and ectopic transformants (Fig. S10).

**Fig 6:**
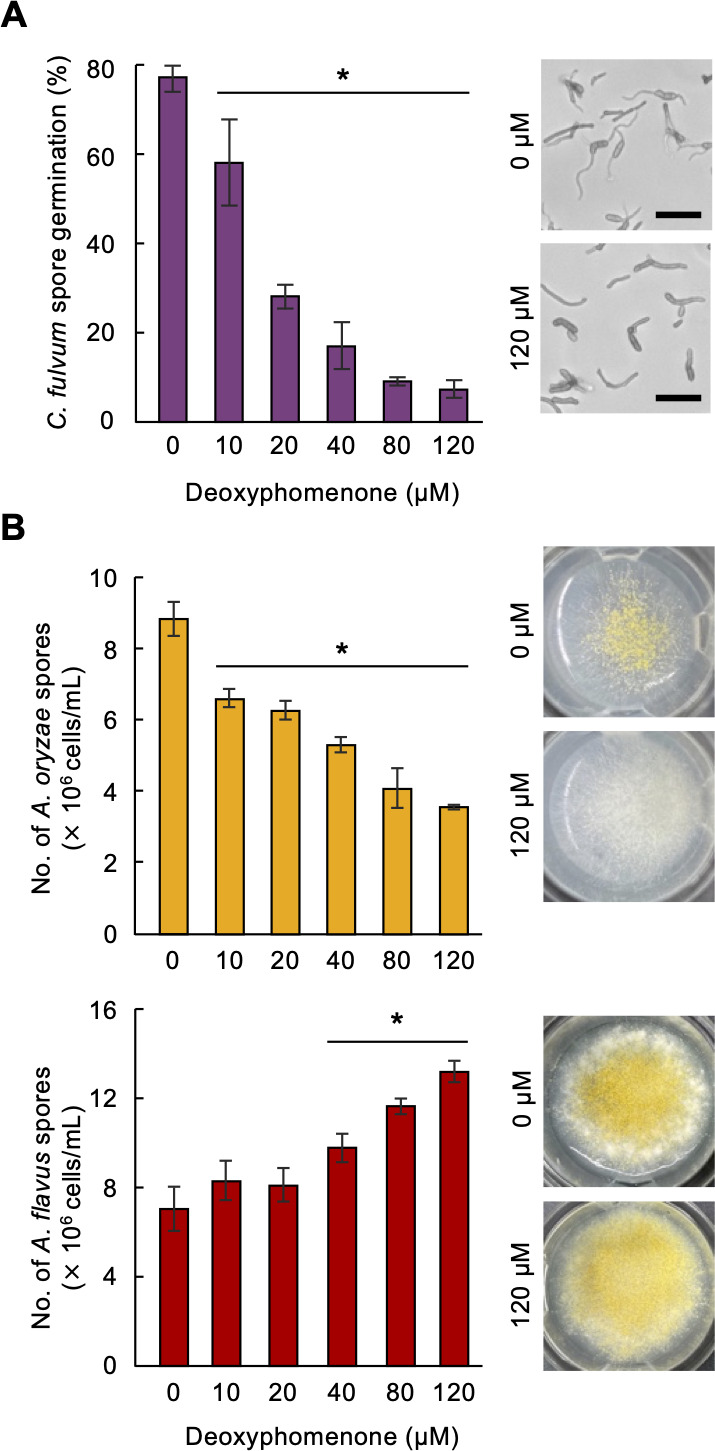
Different effects of deoxyphomenone on spore germination of *Cladosporium fulvum* and spore production of two *Aspergillus* species. Wild-types *C. fulvum* CF301, *A. oryzae* RIB40, and *A. flavus* NBRC114564 were grown on MM agar supplemented with deoxyphomenone. (**A**) Inhibition of spore germination of *C. fulvum*. (**B**) Effects on sporulation of RIB40 and NBRC114564. Values are the mean of three biological replicates. Error bars indicate standard deviations. Asterisks indicate significant difference (*P* < 0.025) compared to control treatment (0 µM) by Williams' test.

We then studied the phenotype of the fungal strains after exposure to deoxyphomenone on MM. Deoxyphomenone was fungistatic against *C. fulvum* over a wide range of concentrations; inhibition by deoxyphomenone was concentration-dependent; and inhibition of spore germination of *C. fulvum* was around 25% at 10 µM, reaching about 90% at 80 µM (Fig. 6A). Thus, the concentration of deoxyphomenone secreted by strain 414-3 seems sufficient to inhibit *C. fulvum* spore germination. As Tanaka et al. (16) treated *A. oryzae* strains with higher concentrations of deoxyphomenone (sporogen-AO 1) to test sporogenic activity, we also tested concentrations up to 120 µM against *A. oryzae* RIB40 and *A. flavus* NBRC114564. In *Aspergillus*, deoxyphomenone treatment also affected sporulation in a concentration-dependent manner, but the effect varied among different species: compared to no treatment, treatment with 120 µM deoxyphomenone reduced spore production by RIB40, but increased it in NBRC114564 by ~90% (Fig. 6B). While deoxyphomenone did not affect colony growth or sporulation of *H. pulvinata* strain 414-3, colony morphology and formation of aerial hyphae were altered in *Aspergillus* strains, and colony growth was also slightly inhibited in RIB40, indicating that RIB40 is less tolerant to this sesquiterpene than 414-3 (Fig. 6B; Fig. S11). These results show that deoxyphomenone has quite different activities in different fungi; it has exogenic antifungal activity against the host fungus and affects endogenic sporogenesis in *Aspergillus* species.

### The deoxyphomenone biosynthetic gene cluster was horizontally transferred from an *Aspergillus* ancestor to *H. pulvinata*

**Fig 7:**
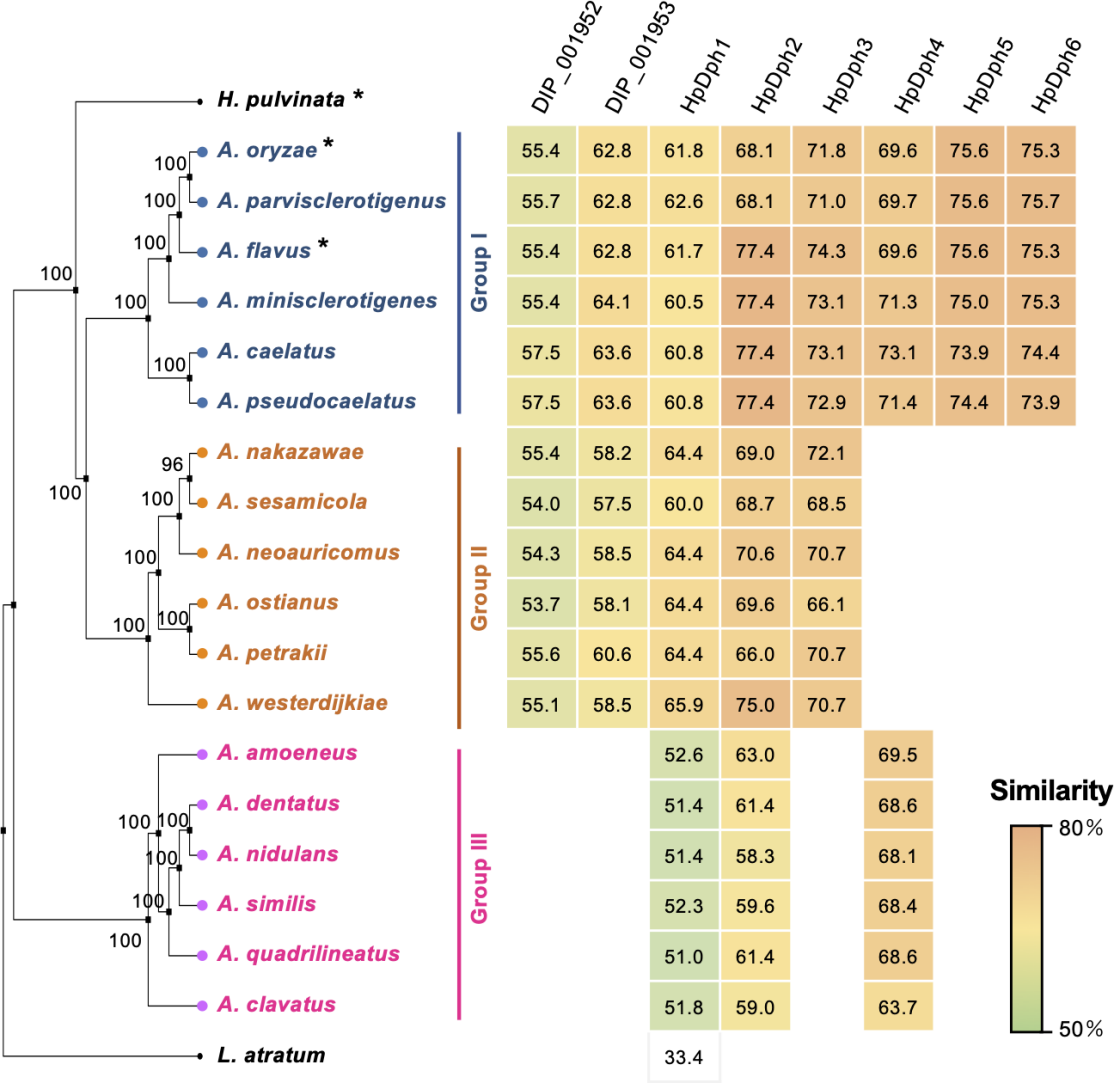
Phylogeny and sequence similarity of deoxyphomenone biosynthetic (*DPH*) genes in *Hansfordia pulvinata* and *Aspergillus* species. The phylogenetic species tree based on the fungal genomic sequences was constructed using maximum likelihood. Numbers at branches represent bootstrap percentages (1,000 replicates). Asterisks represent species that have been verified to produce deoxyphomenone. The sequence of *Lyophyllum atratum* belonging to *Agaricomycetes* was used as an outgroup. *Aspergillus* species were categorized into three groups based on the presence of *DPH* genes. The heatmap represents the pairwise similarity (%) of the deduced amino acid sequences of the *DPH* homologous genes.

We analyzed the commonality and conservation of the *DPH* clusters in genomic sequences of *Aspergillus* species. Complete *DPH* clusters were detected only in *Aspergillus* species from the section *Flavi* (group I; Fig. 2A), and the protein sequences of HpDph2 to HpDph6 in these species were highly conserved, with ~70% pairwise similarity to those of *H. pulvinata* (Fig. 7). Meanwhile, partial *DPH* clusters were present in *Aspergillus* species of the section *Circumdati* (group II: *HpDPH1*, *HpDPH2*, *HpDPH3*) and the sections *Nidulantes* and *Clavati* (group III: *HpDPH1*, *HpDPH2*, *HpDPH4*) (Fig. 7). Group II species also contained a lipase/thioesterase family protein- and integral membrane protein-encoding genes downstream of *HpDPH1*, the same as in Group I (Fig. 7; Fig. S5). *DPH* biosynthesis genes between groups I and II were more similar than those in *H. pulvinata* (Fig. S12). Group III further lacked the commonality and conservation of the *DPH* clusters: the similarities with *HpDPH1* and *HpDPH2* genes were much lower than in the other two groups, the position and the order of *DPH2* and *DPH4* genes in the genome sequence were quite different, and unrelated genes were interspersed in the cluster (Fig. 7; Fig. S5). These results suggested that group II and III species lost parts of the *DPH* cluster and no longer produced deoxyphomenone.

**Fig 8:**
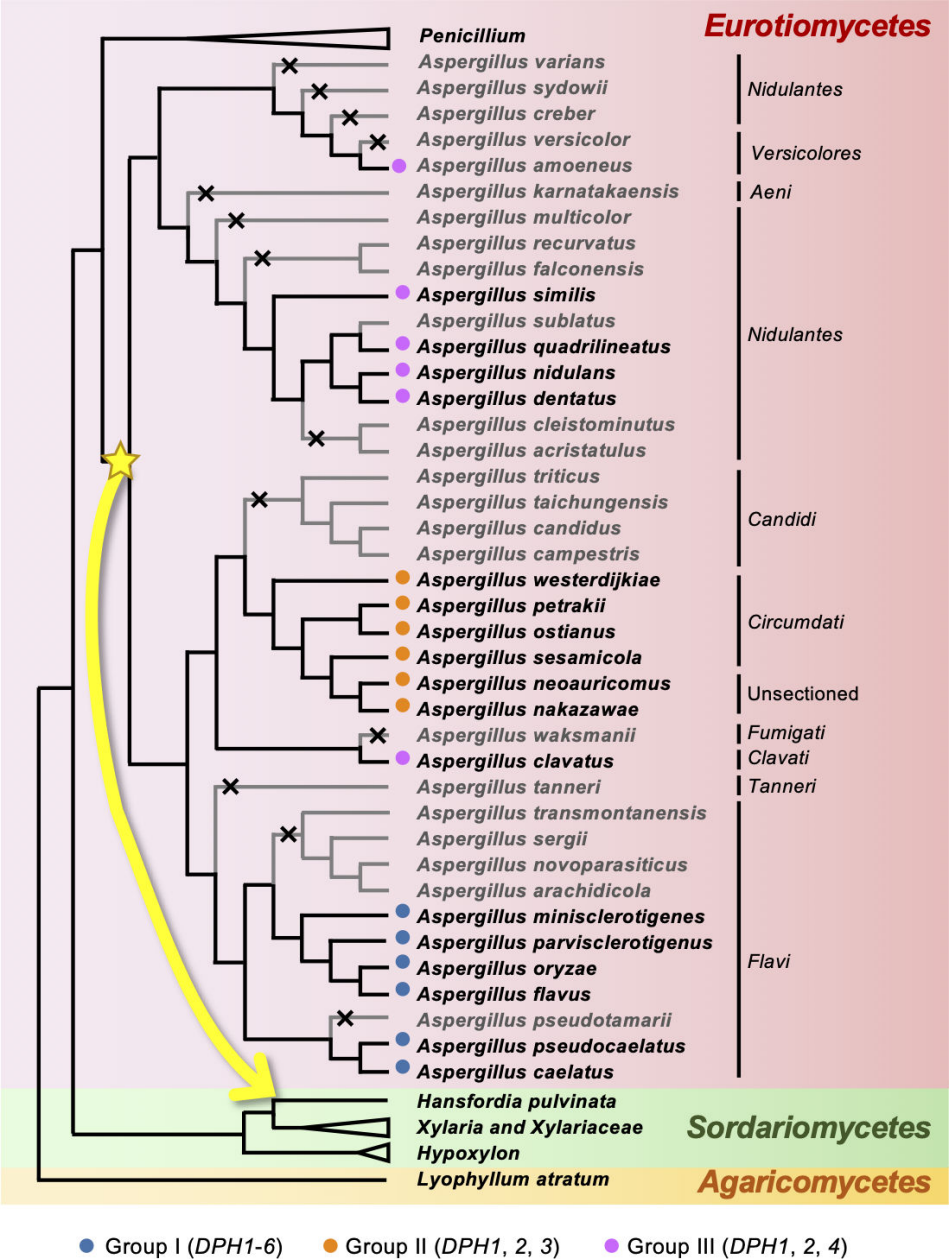
Proposed horizontal transfer of the deoxyphomenone biosynthetic (*DPH*) gene cluster from ancestral *Aspergillus* species to the mycoparasite *Hansfordia pulvinata*. The phylogenetic species tree was based on the coding sequences from 60 fungal genomes and constructed using maximum likelihood with k-strings = 16, and horizontal gene transfer, duplication, and loss events inferred in Fig. S13 were described. Horizontal gene transfer events for the *HpDPH1* (T1) and *DPH2/DPH4* (T2) genes are indicated by yellow stars. Proposed horizontal transfer of the *DPH* cluster from ancestral *Aspergillus* to *H. pulvinata* is highlighted by a yellow arrow. Gene loss is marked by an x on the branch. The three *Aspergillus* groups classified by the presence of *DPH* genes shown are the same as in Fig. 7. Fungal species with either a complete or partial *DPH* cluster are in black, and those without the cluster are in gray. *Aspergillus* sections are given on the right. *Lyophyllum atratum* was used as an outgroup.

To infer the evolutionary origin of the *DPH* clusters in *H. pulvinata* and *Aspergillus*, we analyzed the evolutionary divergence of the clusters among 60 fungal genomes using genomic and phylogenetic approaches with the NOTUNG algorithm, which reconciles differences between species trees and gene trees by inferring gene duplications, transfers, and losses (20). Reconciliation of the species tree with the HpDph1, HpDph2, and HpDph4 protein trees detected a transfer event early in *Aspergillus* (Fig. S13). Likewise, the HpDph3, DIP_001952, and DIP_001953 trees indicated transfer from the ancestor of groups I and II (sections *Flavi* and *Circumdati*) to *H. pulvinata*. The predicted origin of *HpDPH5* and *HpDPH6* genes was only group I (section *Flavi*). Since the *DPH* clusters are highly conserved in *H. pulvinata* and *Aspergillus*, these results suggest an evolutionary scenario, in which the *DPH* cluster was transferred from the common ancestor of groups I and II (sections *Flavi* and *Circumdati*) to *H. pulvinata* at the same time, and that genes were lost in time in *Aspergillus* species (Fig. 8). Since all duplication events detected in *DPH* genes occurred after the horizontal transfer events (Fig. S13), it is unlikely that the duplication is associated with the transfer of the *DPH* cluster. Altogether, these results suggest that the *DPH* cluster arose before speciation within the *Aspergillus* genus. Finally, because we inferred the horizontal transfer of the *DPH* cluster from *Aspergillus* to *H. pulvinata*, we investigated the mycoparasitism of *H. pulvinata* against *Aspergillus*. Contrary to our expectations, *H. pulvinata* 414-3 did not directly penetrate the hyphae of *A. oryzae* RIB40, as observed with the host fungus *C. fulvum*, but physical contact, such as adhesion to or coiling around the hyphae of the RIB40, was detected (Fig. S14).

## DISCUSSION

Strict regulation of expression through the physical linkage of genes involved in the same biosynthetic pathway leads to physiological economization; thus, biosynthetic genes for secondary metabolites in fungi are often maintained in clusters (21), which allows for the rapid conversion of potentially toxic or chemically unstable intermediate products (22). In the present study, the *DPH* clusters were highly conserved between *H. pulvinata* and *Aspergillus* section *Flavi*, suggesting that the final product, deoxyphomenone, plays critical roles in distinct stages of the life cycle of these fungi. Indeed, it acts as an antifungal agent of the mycoparasite *H. pulvinata* against the host fungus *C. fulvum* and controls sporulation at least in *A. oryzae* and *A. flavus*. The correlation of these fungal clusters can be explained through the most parsimonious evolutionary scenario, which is constructed by reconciling the species tree with the *DPH* protein tree in consideration of the costs of evolutionary events (i.e., gene duplications, transfers, and losses) (20). Interestingly, we inferred that the *DPH* cluster was horizontally transferred across a large taxonomic distance from the genus *Aspergillus* (*Eurotiomycetes*) to *H. pulvinata* (*Sordariomycetes*), suggesting that *H. pulvinata* maintained the cluster as deoxyphomenone production to be more conductive to mycoparasitism. Horizontal gene transfer is not rare in fungi (23, 24); gene clusters for the biosynthesis of products, such as fumonisin (25, 26), host-specific toxins in *Alternaria* species (27), and cercosporin (28), have been horizontally transferred across classes, but the final products function in the recipient fungi basically in a similar way.

We deduced the deoxyphomenone biosynthetic pathway from the component genes of the *DPH* cluster in *H. pulvinata* and *Aspergillus*. The bicyclic eremophilane-type sesquiterpene deoxyphomenone is probably formed by the cyclization of farnesyl pyrophosphate catalyzed by sesquiterpene cyclase (17), followed by oxygenation and epoxidation of the double bond in *H. pulvinata* and *Aspergillus*. Although deoxyphomenone has also been detected in *Penicillium* species, which are taxonomically close to *Aspergillus*, the *DPH* cluster was not detected in the published genome sequences of *Penicillium* species; thus, the pathway of deoxyphomenone biosynthesis is likely different between the two genera. On the basis of a relaxed molecular clock analysis, *Aspergillus* and *Penicillium* likely diverged 94.0 mya, with *Aspergillus* sections *Flavi*, *Circumdati*, *Nidulantes*, and *Clavati* originating 76.8 mya (29, 30). Because the *DPH* clusters are fully or partially conserved in *Aspergillus*, we presume that their horizontal transfer to *H. pulvinata* occurred at least before the common ancestor of these sections (Fig. 8).

It is still unclear how *H. pulvinata* acquired the *DPH* clusters from the ancestral *Aspergillus*. Since horizontal transfer of foreign DNA first requires contact between fungal cells, the involvement of anastomosis between conidia, germ tubes, or hyphae and subsequent heterokaryon formation have been proposed (31–33). Since *H. pulvinata* has a wide host range, including plant pathogens (34–39). Hyphae of plant pathogens in *Aspergillus* sections *Flavi* and *Circumdati* might contact with hyphae of *H. pulvinata* on plants and subsequent gene transfer. Mycoparasitic *Trichoderma* species have been suggested to have acquired by horizontal transfer nearly half their genes encoding plant cell wall-degrading carbohydrate-active enzymes and auxiliary proteins enabling them to be parasitic on a broad range of *Ascomycota* (40). *H. pulvinata* 414-3 did not establish a direct parasitic relationship with *A. oryzae* RIB40 as it did with *C. fulvum*, and no distinct anastomosis was observed, but it firmly adhered to and coiled around RIB40 hyphae, indicating basic compatibility facilitating horizontal gene transfer between the two fungi. In the genomic sequence of *H. pulvinata*, a gene cluster derived from *Aspergillus* was found in this study, but no horizontal gene transfer between host fungi, including *C. fulvum*, has been found so far. Since *H. pulvinata* completely kills the host *C. fulvum*, *C. fulvum* is not likely to be a source of foreign genes in horizontal transfer. Rather, *H. pulvinata* might have acquired the gene cluster in direct contact with nonhost fungi, such as *Aspergillus*. Although transduction by mycoviruses or propagation of retrotransposons could also have facilitated horizontal gene transfer (41), no transposon-like sequences were detected around the *DPH* cluster.

Several environmental cues, such as temperature, light, nutrients, pH, and competing or synergistic organisms, influence fungal activity, including transcriptional regulation of secondary metabolism-associated gene clusters (42). Aflatoxin production in *A. flavus* is generally upregulated at a suboptimal 30°C (43). Similarly, we found that deoxyphomenone production was also highest at temperatures unsuitable for growth of *H. pulvinata* and *Aspergillus* species (Fig. S6). In *A. flavus*, Δ*AfDPH1* mutants that no longer produced deoxyphomenone formed significantly less spores. These results suggest that in at least *A. flavus*, deoxyphomenone stimulates sporulation at unsuitable temperatures. In addition, *A. flavus* strains tended to produce more deoxyphomenone than *A. oryzae* strains, but the level was not sufficiently high for antifungal activity. Gibbons et al. (44) reported that the expression levels of many secondary metabolite gene clusters of *A. oryzae*, including those for *DPH* and aflatoxin, were repressed compared with those of *A. flavus. A. oryzae* used to ferment rice to produce Japanese sake is probably derived from *A. flavus*, a pathogen of plants and animals (44). *A. oryzae* is cultured with the budding yeast *Saccharomyces cerevisiae* during sake brewing and might have lost the ability to produce aflatoxin during its domestication from *A. flavus* (45) because aflatoxin is genotoxic to yeast (46). Since *A. oryzae* is usually grown under optimal fermentation conditions, the gene encoding deoxyphomenone required for sporulation by *A. flavus* in unfavorable conditions may also have been silenced or lost in *A. oryzae*. In fact, deoxyhomenone was not detected in the three *A. oryzae* strains, and external application of deoxyphomenone inhibited sporulation and colony formation only in *A. oryzae*, indicating that *A. oryzae* RIB40, which produces only low levels of the compound, is less tolerant to this sesquiterpene (Fig. S11). The mycoparasite *H. pulvinata* also produced deoxyphomenone at temperatures unfavorable for its growth, but in the presence of its host fungus *C. fulvum*, expression of the *HpDPH* genes was strongly induced. However, the *ΔHpDPH1* mutant strains of *H. pulvinata* parasitized *C. fulvum*, comparable to the wild-type strain, supporting that deoxyphomenone is not required directly for its mycoparasitism. Spore germination of *C. fulvum* treated with deoxyphomenone for 24 h was halved, also suggesting an epidemiological effect on the mycoparasitism of *H. pulvinata*.

## MATERIALS AND METHODS

A detailed description of the materials and methods is included in the supplemental text.

### Growth conditions for fungal strains and plants

Strains of *H. pulvinata* 414-3 and *C. fulvum* CF301 were grown on PDA (half-strength, BD Difco, USA), MM agar or broth (15 g sucrose, 5 g ammonium tartrate, 1 g NH_4_NO_3_, 1 g KH_2_PO_4_, 0.5 g MgSO_4_⋅7H_2_O, 0.1 g NaCl, 0.1 g CaCl_2_⋅H_2_O, 25 µL 0.2 mg/mL biotin, 15 g agar and 1 mL trace elements per liter) at 25°C in the dark for 1 and 2 weeks, respectively, and then spores were collected in sterile distilled water. *A. oryzae* and *A. flavus* strains were cultured on PDA, MM agar and broth, and Czapek Dox agar and broth (3 g NaNO_3_, 2 g KCl, 1 g KH_2_PO_4_, 0.5 g MgSO_4_⋅7H_2_O 20 g glucose and 15 g agar per liter adjusted to pH 6.5) at 30°C in the dark for a week, and spores were collected in sterile distilled water containing 1% (vol/vol) Tween 20.

Tomato cultivar Moneymaker was grown in a climate chamber at 25°C with 16 h light/8 h dark for 3 weeks. The lower sides of tomato leaves were sprayed with a spore suspension of *C. fulvum* CF301 (1 × 10^5^ spores/mL). After 2 weeks, a spore suspension of *H. pulvinata* 414-3 (1 × 10^5^ spores/mL) was sprayed on the brown lesions that had formed on the abaxial surfaces of the leaves. White mycelial patches of *H. pulvinata* were observed a week after inoculation.

### Electron microscopy

Fungal strains of *C. fulvum* (CF301 and KO10) and *A. oryzae* RIB40 were cultured on a nylon membrane placed on PDA in a petri dish and incubated as described above. The membranes were then transferred to water agar and incubated for a week in the same conditions. A spore suspension of *H. pulvinata* (1 × 10^5^ spores/mL) was sprayed on the test colony on the membrane, then white colonies were observed 1 week later with a SU-8220 scanning electron microscope (Hitachi, Tokyo, Japan) as previously described (9).

Spores of strain CF301 were treated with deoxyphomenone (120 µM) at 25°C for 24 h in the dark. Spores collected by centrifugation were fixed as described in the supplemental text. Ultrathin sections were cut using an ultramicrotome UCT Ultracut (Leica, Wetzlar, Germany), then stained with uranyl acetate and observed with a TEM H-7600 (Hitachi).

### Assays of antifungal activity and plant toxicity of deoxyphomenone

*C. fulvum* spores were treated with deoxyphomenone (sporogen-AO1; Apollo Scientific, UK) in 1% methanol (vol/vol) at 25°C in the dark for 24 h and observed using a Nikon E600 light microscope (Tokyo, Japan).

The antifungal activity of deoxyphomenone was compared with that of an *N*-halo-alkylthioimide fungicide, catn (orthocide80; Arysta Life Science, Tokyo, Japan). *C. fulvum* spores (1 × 10^3^ cells/mL) were treated with distilled water, deoxyphomenone (120 µM), or captan (100 µM), then each solution was replaced with fresh solution, and samples were incubated another 24 h. Germinated spores were then counted using the Nikon light microscope. For the controls, dH_2_O or 1% methanol (vol/vol) was used. Each treatment was done in triplicate, and at least 200 spores and hyphae were assessed for each treatment.

Spore suspensions of the strains *C. fulvum* CF301, *P. fuligena* Pf17923, and *F. oxysporum* f. sp. *lycopersici* CK3-1 were adjusted to 1 × 10^6^ spores/mL, and 10 µL was dropped on MM agar supplemented with different concentrations of deoxyphomenone (20 to 120 µM). The strains were cultured at 25°C in the dark.

### Phylogenetic analyses of deoxyphomenone biosynthetic gene clusters

Sequences shown in Table S2 were aligned using MAFFT online version 7 (47) with default parameters and trimmed using TrimAl version 1.4 (48). Maximum likelihood phylogenetic trees were constructed using RAxML version 8.2.12 with 1000 bootstraps; the PROTCATAUTO model was selected for amino acid sequences (49). Pairwise sequence alignments of *DPH6* homologous genes were performed using EMBOSS Needle (50).

The phylogenetic species tree was constructed using maximum likelihood based on the 500 monocore genes (a single homolog in each of the species) using CVtree version 3.0 (*k* = 16) (51) by uploading the genomic information for the 60 published fungi shown in Table S1.

Reconciliation analysis between species and gene trees was performed using NOTUNG v.2.9 (20) according to the instructions (version 2.8 beta) to infer the evolutionary trajectories of the *DPH* clusters under every possible rooting. Event scores were calculated as the total costs of duplications, transfers, and losses. Costs/weights were set as duplications (D), 1.5; transfers (T), 8.0; losses (L), 1.0 (ratio D:T:L is 1:5.3:0.67) for amino acid sequences, and D, 1.5; T, 6.0; L, 1.0 (ratio D:T:L is 1:4:0.67) for nucleotide sequences for the gene clusters.

### Quantitative detection of deoxyphomenone

*H. pulvinata* (1 × 10^6^ spores) was cultured in 50 mL of MM broth with shaking at 25°C in a light/dark cycle of 16 h/8 h for 2 weeks. *A. oryzae* and *A. flavus* (1 × 10^6^ spores) were incubated in MM broth without shaking at 25°C in the dark for 2 weeks. Deoxyphomenone in the culture supernatants was quantified using a 4000 QTRAP LC–MS/MS System (Sciex, MA, US) as previously reported (9). Commercial deoxyphomenone (Apollo Scientific) was used as a standard.

### Construction of transformation vectors

The upstream and downstream regions of *HpDPH1*, *AoDPH1*, and *AfDPH1* genes amplified from each genomic DNA and hygromycin resistance gene (*hph*) and pyrithiamine-resistant gene (*ptrA*) cassettes were inserted into the plasmid pPM43GW or pSH75 (52) using the In-Fusion EcoDry Cloning Kit (TakaraBio) (Fig. S8) as described in the supplemental text. *AoDPH1* (without introns) was driven under the promoters of the *A. nidulans trpC* gene and inserted into the blunt end of the PvuII-digested plasmid pPZP-PvuII.

Plasmid vectors were introduced into *Escherichia coli* DH5α (NIPPON GENE, Tokyo, Japan) and extracted using MagExtractor -Plasmid- (TOYOBO) according to the instructions. The correct orientation of the fragments in the final constructs was confirmed by PCR.

### Fungal transformation

*A. tumefaciens*-mediated transformations were performed as previously described (53). The transformants were selected on PDA plates supplemented with G418 (Fujifilm Wako, Osaka, Japan) or hygromycin (Fujifilm Wako) in the dark at 25°C. Gene replacement was confirmed by PCR amplification using the primers listed in Table S3.

Protoplasts of *A. oryzae* RIB40 and *A. flavus* NBRC114564 were transformed using polyethylene glycol as described in the supplemental text. The transformants were selected on Czapek Dox agar containing 0.2 µg/mL pyrithiamine hydrobromide. Gene replacement of the transformants was confirmed by PCR amplification using the primers listed in Table S3.

### Quantitative real-time PCR

Quantitative real-time PCR was performed using designed primers (Table S3) as previously described (54). Relative expression levels were calculated using the comparative CT (2^−∆∆CT^) method (55). The data were normalized to the transcript level of the *H. pulvinata* actin gene (Table S3). Transcript levels of target genes in each RNA sample were measured for three independent experiments, each with two replicates.

### Statistical analyses

Significant differences (*P* < 0.05) of *C. fulvum* and *Aspergillus* strains were evaluated using either Tukey’s test or Williams’ multiple comparison. Significant differences (*P* < 0.05) in gene expression levels were determined using Welch’s *t*-test, followed by Bonferroni–Holm correction for multiple testing. All data were analyzed in the program R version 4.0.3 (https://www.r-project.org/).

## Data Availability

The genomic sequence of *H. pulvinata* 414-3 is available in the DDBJ repository under accession number BJKQ00000000 (BioProject: PRJDB8178). Other fungal sequences shown in Table S1 are available on the fungal genome portal MycoCosm (mycocosm.jgi.doe.gov) of the Joint Genome Institute (Lawrence Berkeley National Institute, Berkeley, CA, USA) (19) and in the DDBJ/EMBL/GenBank repository (https://www.ncbi.nlm.nih.gov/). Auxiliary data are available through Figshare (https://doi.org/10.6084/m9.figshare.24559120).

## SUPPLEMENTAL MATERIAL

Supplemental figures mbio.04007-24-s0001.pdf Fig. S1 to S14.

Supplemental text mbio.04007-24-s0002.docx Supplemental methods.

Supplemental tables mbio.04007-24-s0003.xlsx Table S1 to S3.

ASM does not own the copyrights to Supplemental Material that may be linked to, or accessed through, an article. The authors have granted ASM a non-exclusive, world-wide license to publish the Supplemental Material files. Please contact the corresponding author directly for reuse.
