# Supplementary material for: Adaptive evolution of sesquiterpene deoxyphomenone in mycoparasitism by Hansfordia pulvinata associated with horizontal gene transfer from Aspergillus species
Source: mBio. 2025 Mar 20;16(4):e04007-24. doi: 10.1128/mbio.04007-24 (PMC11980549; doi:10.1128/mbio.04007-24)
Supplement: Supplemental figures — Fig. S1 to S14. [file mbio.04007-24-s0001.pdf]

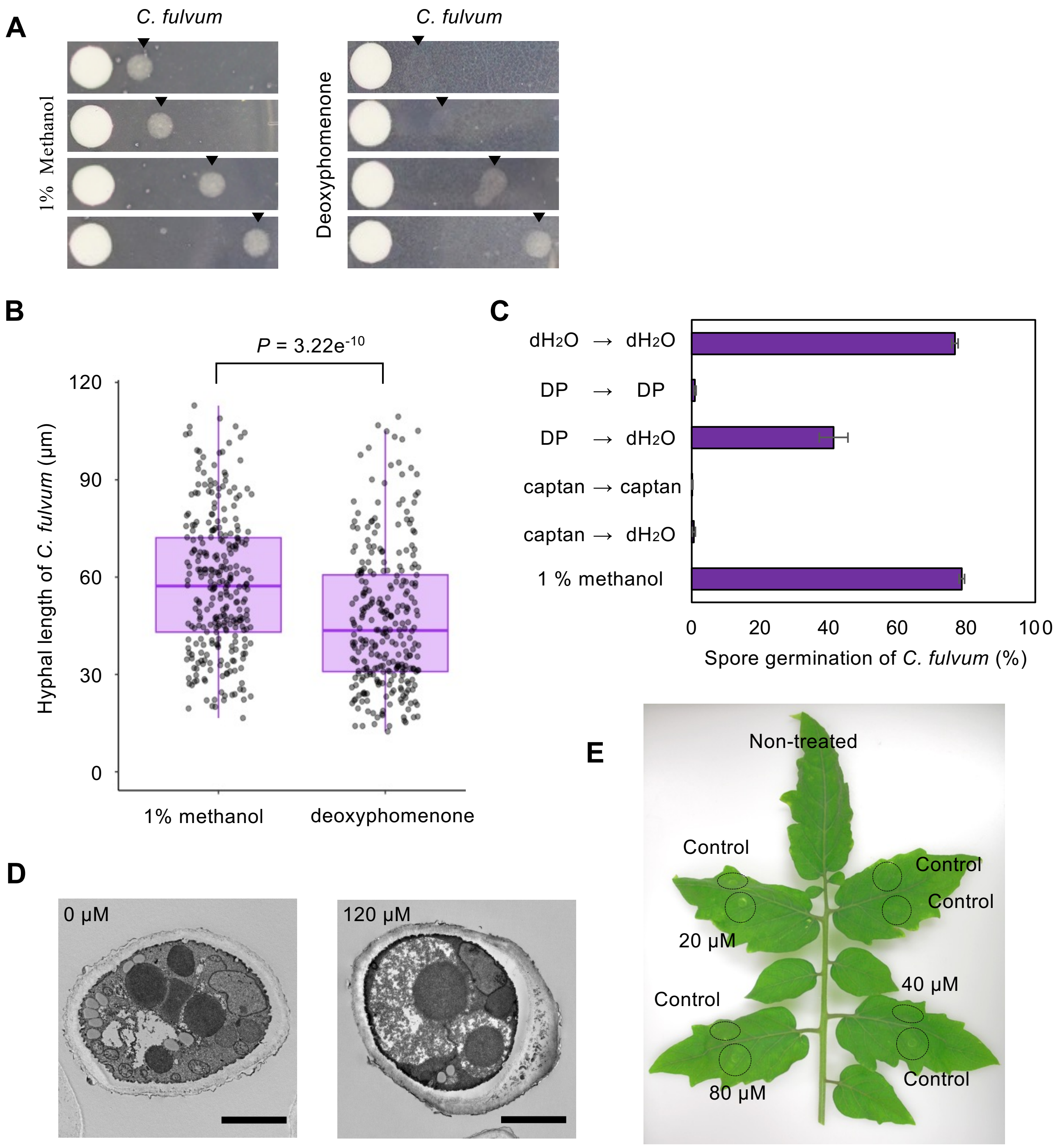

Fig. S1. Effect of deoxyphomenone on *Cladosporium fulvum* and tomato leaves.
Deoxyphomenone (120 µM) and 1% methanol (v/v) as a control were used. (**A**) Effect on spore germination. A filter paper containing deoxyphomenone was placed on the left on PDA, then a spore suspension of *C. fulvum* was dropped (arrowheads) at different distances from the paper and incubated at 25 ºC for 2 weeks. (**B**) Effect on hyphal elongation. Spores were germinated in a sterile distilled water at 25 ºC for 24 h. Germinated spores were treated with deoxyphomenone, then hyphal length was measured after 24 h using a light microscope. The line in the center of the box indicates the median; box margins represent the 25th and 75th percentiles. The length of the box is the interquartile range; whiskers indicate the minimum and maximum. Significant differences among treatments were determined using Tukey's test. (**C**) Fungistatic activity of deoxyphomenone. Spores of *C. fulvum* were suspended in distilled water (dH2O), deoxyphomenone (DP) or 100 µM fungicide captan for 24 h, then solutions were replaced with fresh solutions as shown, and treated for a further 24 h. Germinated spores were counted using a light microscope. Continuous treatment with dH2O or 1% methanol (v/v) was used as a positive control. Values are the means of three replicates (± SD). (**D**) Transmission electron micrographs of interior of *C. fulvum* spores 24 h after treatment with deoxyphomenone. Bars = 2 µm. (**E**) Evaluation of 20, 40 and 80 µM deoxyphomenone or 1% methanol (v/v) as a control for toxicity on tomato leaves 2 weeks after 1-month-old leaves were injected with the compound as described in Supplemental Methods.

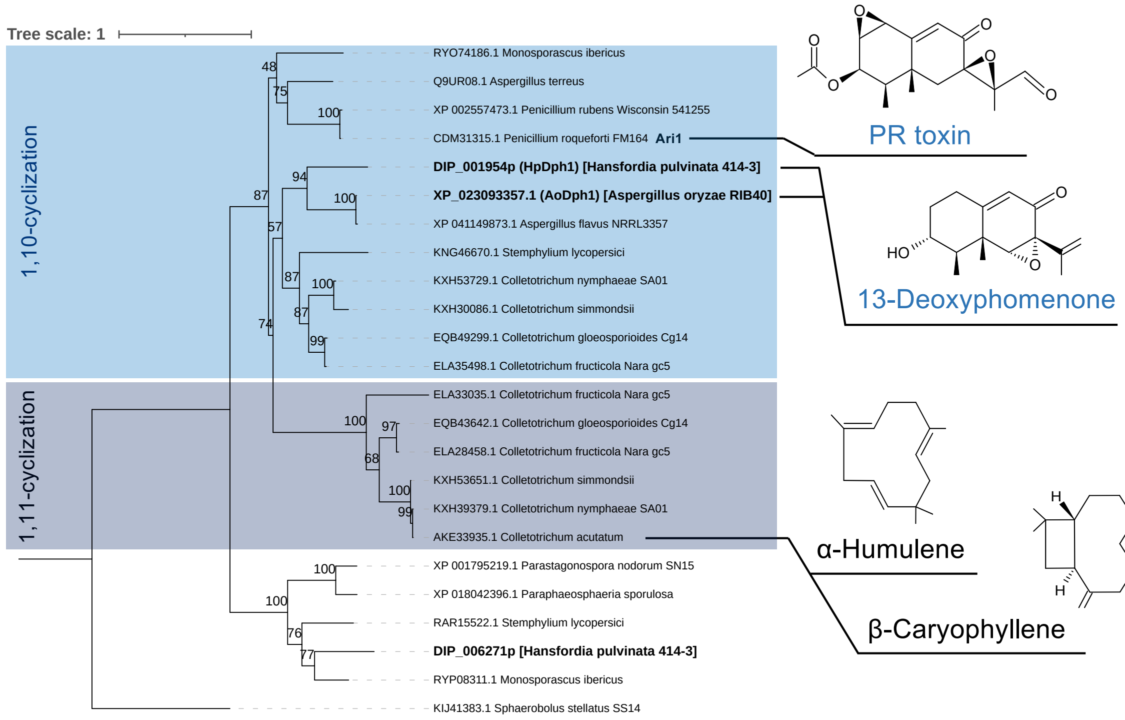

Fig. S2. Phylogenetic tree of fungal sesquiterpene cyclases.

The phylogenetic tree was generated from amino acid sequences encoding sesquiterpene synthases by maximum likelihood phylogenetic analysis. Predicted aristolochene synthase-like proteins identified in *H. pulvinata* 414-3 and *A. oryzae* RIB40 genome sequences, and the aristolochene synthase Ari1 are shown in bold. GenBank/EMBL/DDBJ accession numbers or unique numbers for *H. pulvinata*are are indicated. *Sphaerobolus stellatus* (*Basidiomycota*) was used as an outgroup. Numbers beside the branches represent bootstrap values based on 1000 replicates. The scale bar corresponds to 0.1 estimated amino acid substitutions per site. Representative metabolites and proteins associated with their cyclization are connected by lines.

```
DIP-001954p(HpDPH1)    1 MLSTLRSFELHKLLGLTGSSSNDSTTTTTTITTTAASAAPQPKAQQQPQP    AYPSESEDMARDRRRFPSLAATTDTASPSPSAFAPEIHPAADRVSREVAE   100
                                                .......::|:|.....||.:...|:.    ..      :.:|||..|.||    ..:|.||||:..|||.:|.||.||..
XP_023093357(AoDPH1)   1 -----------------------MLQRLWALSTSAIKLPFQPFSFGAPRD    LL------IEKDRRSMPCLA----KEAPPPSAFSATIHPLSDSVSTEVDN    67

DIP-001954p(HpDPH1)  101 AFLARWPFRGEAERRRFRAADFPRVTCLYFPHARAERIGFACRLLTLLFL    VDDLLEEMGLEEGSAYNERLISISKGDEEPEPDKPAEVLTRELWEDMRAC   200
                          .||..||||.:.||.||.||.|.||||||||.|..:|||||||:||:|||    :|||||||.|:|||.|||:|||||:||..|:...||:.:..:|||||||
XP_023093357(AoDPH1)  68 YFLQNWPFRTDNERARFHAAGFSRVTCLYFPMAMDDRIGFACRMLTILFL    IDDLLEEMSLDEGSTYNEKLISISRGDVAPDRTIPAQWIMYDLWEDMRAC   167

DIP-001954p(HpDPH1)  201 DARLAGEIKEPVFTFMRAQTDGSRLTLRELGAYFEYREKDVGQA------    ----LLSALMRFSMALHLTPAQLRLAQPLERNCARHISVVNDIYSWRKEV   290
                         |..||.|:.||||||||||||.:|||:.:.|.|.:|||||||||              |||.|.|::|.|:||...||:|.|.|||||:||:::|||||||||:
XP_023093357(AoDPH1) 168 DHVLADELLEPVFTFMRAQTDKTRLTIHQFGEYLDYREKDVGQAIKAQTK    CIRSLLSGLQRYTMKLYLTEEDLRMAAPAERNCAKHIAILNDIYSWRKEL   267

DIP-001954p(HpDPH1)  291 RASETLHEEGAALCSSVAVLAAEASVPASGARRVLWALCREWEAEHRRME    GDLLRRLVEEEEEEGDQGGQVLRVYVRGLESQMSGNERWSESTPRYHKVK*   391
                         .||:|||.||||:||||.||:...::.:.:.:||||.:|||||:.|:::              ..|....|.:.|..|:.|||.|||||||||||||||..
XP_023093357(AoDPH1) 268 LASKTLHHEGAAICSSVQVLSEVTALSHAATQRVLWTMCREWESVHKQL-    ----------VTEVAGTGSRDLLDYIHGLEFQMSGNERWSESTPRYHF*--   355
```

Identity: 197/401 (49.1%) / Similarity: 247/401 (61.6%) / Gaps: 56/401 (14.0%)

```
DIP-001954p(HpDPH1)    1 -----------MLSTLRSFELHKLLGLTGSSS-----NDSTTTTTTITTT    AASAAPQPKAQQQPQPAYPSESEDMARDRRRFPSLAATTDTASPSPSAFA    84
                                    .|||:.|  |.:|..:...||     |.|:..:.|..||    |.:..|.|:..|:|                           |...|:...
DIP-006271p            1 MASALLSLPTAALSTILS--LVRLSSVVSPSSTITTKNPSSNASATEETT    ATTPPPVPENSQRP--------------------------AGLRPTRLT    71

DIP-001954p(HpDPH1)   85 PEIHPAADRVSREVAEAFLARWPFRGEAERRRFRAADFPRVTCLYFPHAR    AERIGFACRLLTLLFLVDDLLEEMGLEEGSAYNERLISISKGDEEPEPDK   184
                         ...|...::...:|.:.||..|||:.:..||||....:....|:..|.:.    .|||.:.|||||:.||:|||::.|.:.||:|:|..::...:|.:.|:.|.
DIP-006271p           72 ARKHRLTEQTVEQVNDFFLRNWPFKTDKHRRRFVDEGYAFFVCVLVPESL    DERIHWGCRLLTVGFLIDDLVDNMNVAEGAAFNAAVVECCRGTQLPDRDV   171

DIP-001954p(HpDPH1)  185 PAEVLTRELWEDMRACDARLAGEIKEPVFTFMRAQTDGSRLTLRELGAYF    EYREKDVGQALLSALMRFSMALHLTPAQLRLAQPLERNCARHISVVNDIY   284
                         |::.:..:|:|.|||.|.|||.|:.:|...|:.||.||||.....|..||    |||:.|:|:.|:|.:|||...|.:|.|:|.:.:|::.|..:||:.|||:.
DIP-006271p          172 PSQWIMYDLFEAMRAVDRRLADELLQPTIDFLLAQVDGSRRRPMNLAEYF    EYRDADLGKGLISGIMRFCGGLSMTTAELDVVRPVDENVMKHITFVNDVC   271

DIP-001954p(HpDPH1)  285 SWRKEVRASETLHEEGAALCSSVAVLAAEASVPASGARRVLWALCREWEA    EHRRMEGDLLRRLVEEEEEEGDQG-GQVLRVYVRGLESQMSGNERWSEST   383
                         |:.||..|:|..:|.| .:||||.::||...|....|....:||:|...|.||.    .|..|:.|:|         .|..| ...||.|:|.:|.|.|||||.||..|
DIP-006271p          272 SYEKERLAAEAGYELG-EICSSVPIVAAWLGVGEDDAKRVMWQAARGWED    RHLAMKRDIL---------AGPLGASSALRTYLRWVEYQASGNELWSLLT   361

DIP-001954p(HpDPH1)  384 PRYHKVK*------------    391
                         |||::...
DIP-006271p          362 PRYNRFGALGFTEGRPEAQ*    381
```

Identity: 140/420 (33.3%) / Similarity: 207/420 (49.3%) / Gaps: 68/420 (16.2%)

```
XP_023093357(AoDPH1)   1 MLQRLWALSTSA-------IKLPFQPFSFGAPRDLLIEKDRRSMPCLAKE    ----APP-------------PSAFSATIHPLSDSVSTEVDNYFLQNWPFR    76
                         |...|.:|.|:|       ::|.    |..:|...:..|:..|.....:|       .||             |:..:|..|.|::....:|:::||:||||:
DIP-006271p            1 MASALLSLPTAALSTILSLVRLS----SVVSPSSTITTKNPSSNASATEE    TTATTPPPVPENSQRPAGLRPTRLTARKHRLTEQTVEQVNDFFLRNWPFK    96

XP_023093357(AoDPH1)  77 TDNERARFHAAGFSRVTCLYFPMAMDDRIGFACRMLTILFLIDDLLEEMS    LDEGSTYNEKLISISRGDVAPDRTIPAQWIMYDLWEDMRACDHVLADELL   176
                         ||..|.||...|::...|:..|.::|:||.:.||:||:.||||||::.|:    :.||:.:|..::...||...|||.:|:|||||||:|.|||.|..||||||
DIP-006271p           97 TDKHRRRFVDEGYAFFVCVLVPESLDERIHWGCRLLTVGFLIDDLVDNMN    VAEGAAFNAAVVECCRGTQLPDRDVPSQWIMYDLFEAMRAVDRRLADELL   196

XP_023093357(AoDPH1) 177 EPVFTFMRAQTDKTRLTIHQFGEYLDYREKDVGQAIKAQTKCIRSLLSGL    QRYTMKLYLTEEDLRMAAPAERNCAKHIAILNDIYSWRKELLASKTLHHE   276
                         :|...|:.||.|.:|.......||.:||:.|:|          :.|:||:    .|:...|.:|..:|.:..|.:.|..|||..:||:.|:.||.||::. .:|
DIP-006271p          197 QPTIDFLLAQVDGSRRRPMNLAEYFEYRDADLG----------KGLISGI    MRFCGGLSMTTAELDVVRPVDENVMKHITFVNDVCSYEKERLAAEA-GYE   285

XP_023093357(AoDPH1) 277 GAAICSSVQVLSEVTALSHAATQRVLWTMCREWESVHKQLVTEV-AGT--    GSRDLLDYIHGLEFQMSGNERWSESTPRYHF*--------------    355
                         ...|||||.:::....:.....:||:|...|.||..|..:.:.:: ||.    .|..|..|:..:|:|.||||.||..||||:..
DIP-006271p          286 LGEICSSVPIVAAWLGVGEDDAKRVMWQAARGWEDRHLAMKRDILAGPLG    ASSALRTYLRWVEYQASGNELWSLLTPRYNRFGALGFTEGRPEAQ*    381
```

Identity: 134/396 (33.8%) / Similarity: 205/396 (51.8%) / Gaps: 56/396 (14.1%)

Fig. S3. Alignment and homology of the amino acid sequences of the predicted sesquiterpene cyclases identified in *Hansfordia pulvinata* and *Aspergillus oryzae* genome sequences.

Amino acid sequences of two candidates of *H. pulvinata* 414-3, DIP_001954 (HpDph1) and DIP_006271, and one of *A. oryzae* RIB40 XP_023093357 (AoDph1) were compared.

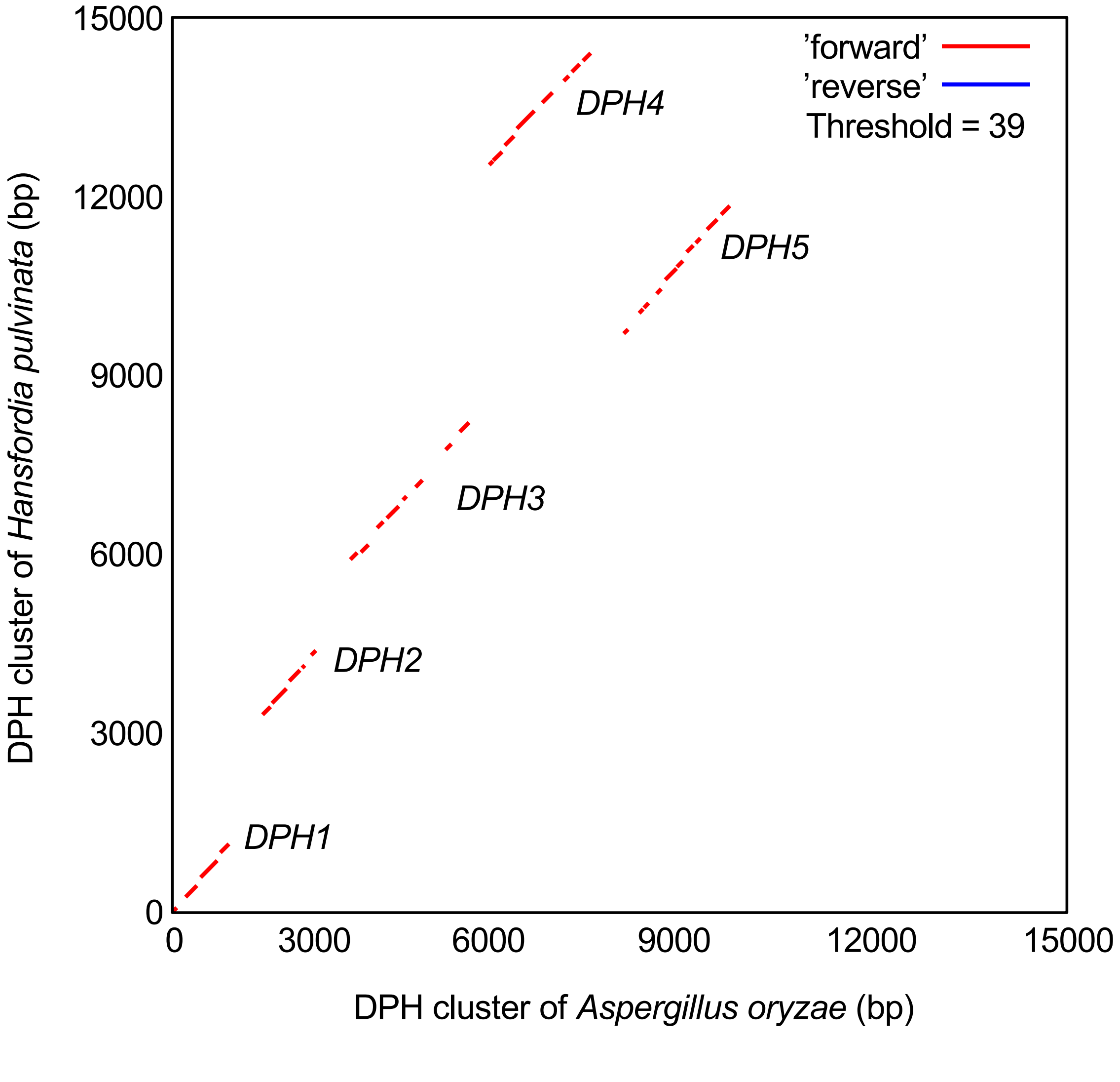

Fig. S4. Dot plot analysis of the deoxyphomenone biosynthesis (*DPH*) gene clusters from *Hansfordia pulvinata* 414-3 and *Aspergillus oryzae* RIB40.

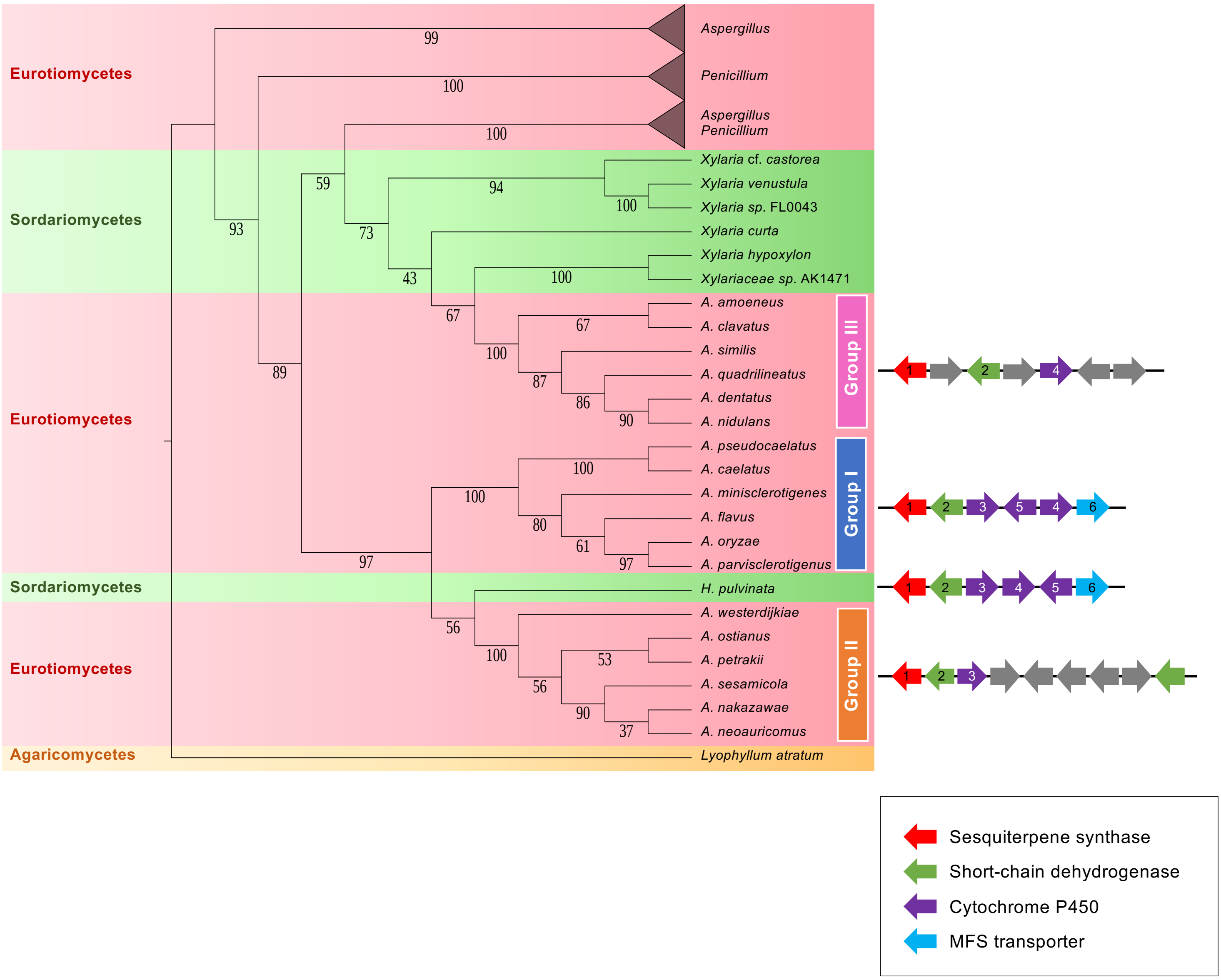

Fig. S5. Phylogenetic analysis of amino acid sequences homologous to HpDph1 using the maximum likelihood method.

Amino acid sequences homologous to HpDph1 from the 58 fungi listed in Table S1 are available in the Figshare. Numbers at branches represent bootstrap percentages (1000 replicates). The sequence of *Lyophyllum atratum* belonging to *Agaricomycetes* was used as an outgroup. Schematic diagrams of genomic structures on the right indicate gene orientation with annotation information in color; numbers in the arrows indicate genes that are more than 50% homologous to HpDph1 to HpDph6 proteins of *H. pulvinata*.

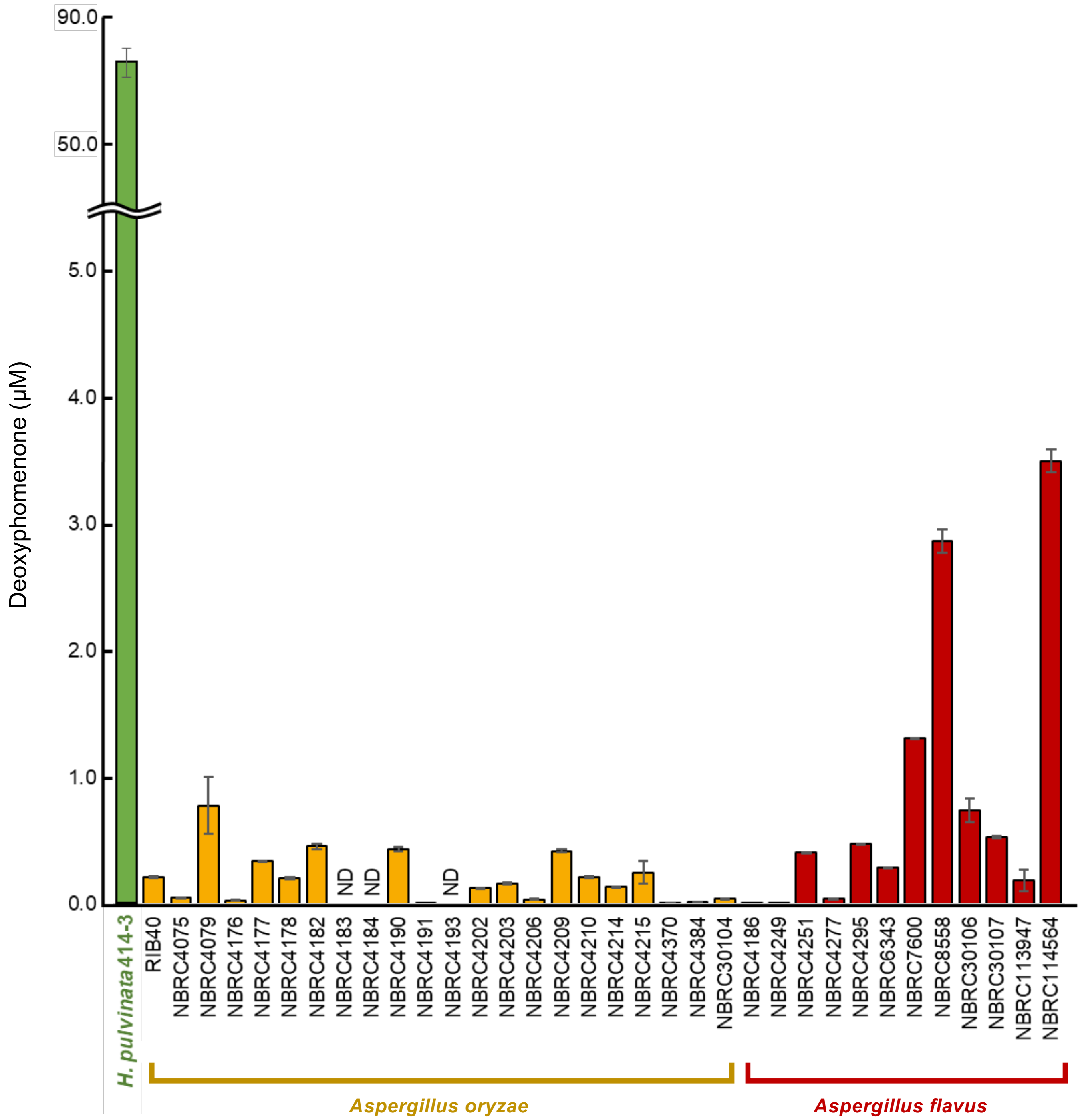

Fig. S6. Deoxyphomenone produced by *Hansfordia pulvinata* 414-3 and strains of *Aspergillus oryzae* and *A. flavus*.

Strains were cultured in MM broth at 25 ºC. Deoxyphomenone in the culture filtrate was quantified using LC-MS/MS. Values are the means of three replicates (± SD). ND, not detected.

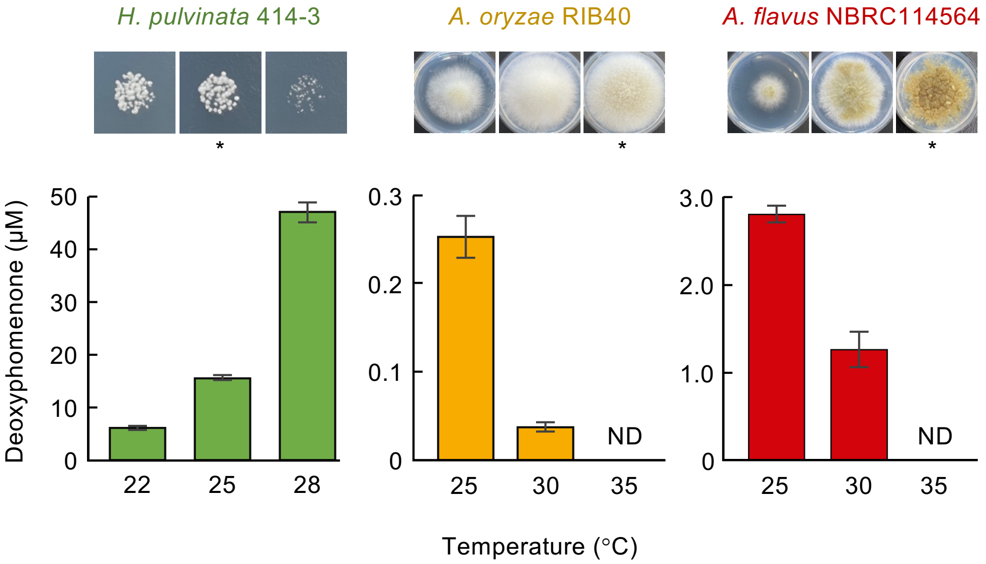

Fig. S7. Deoxyphomenone production by *Hansfordia pulvinata* 414-3, *Aspergillus oryzae* RIB40 and *A. flavus* NBRC114564 on MM agar or in broth at different temperatures.

Deoxyphomenone in the culture filtrate of the broth was quantified by LC-MS/MS. The optimum temperature for growth on agar is indicated by the asterisk. Values are the means of three replicates (± SD). ND, not detected.

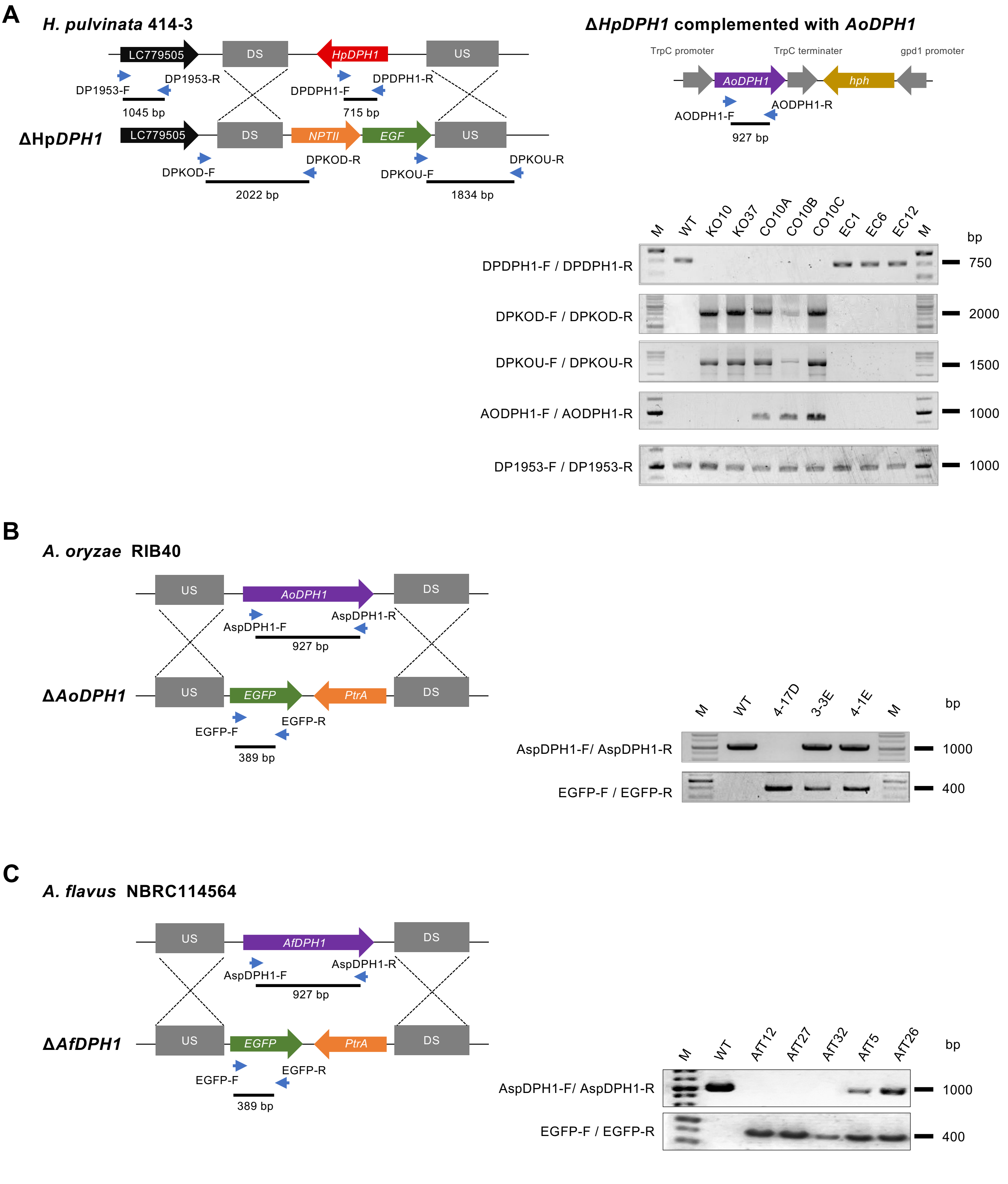

Fig. S8. PCR detection of the inserted cassettes in transformants and wild-type strains.
Representation of the locus of *HpDPH1* homologous genes in wild-type strains of *Hansfordia pulvinata* 414-3 (A), *Aspergillus oryzae* RIB40 (B), *A. flavus* NBRC114564 (C) and transformants. Target genes were replaced by homologous recombination of the downstream (DS) and upstream (US) regions. *HpDPH1* knock-out mutant strain KO10 of *H. pulvinata* was complemented with functional *AoDPH1*. Large arrows: gene sequence; small blue arrows: primer sequence. The expected sizes of the amplicons are indicated under the primers. Each amplicon is shown on the right.

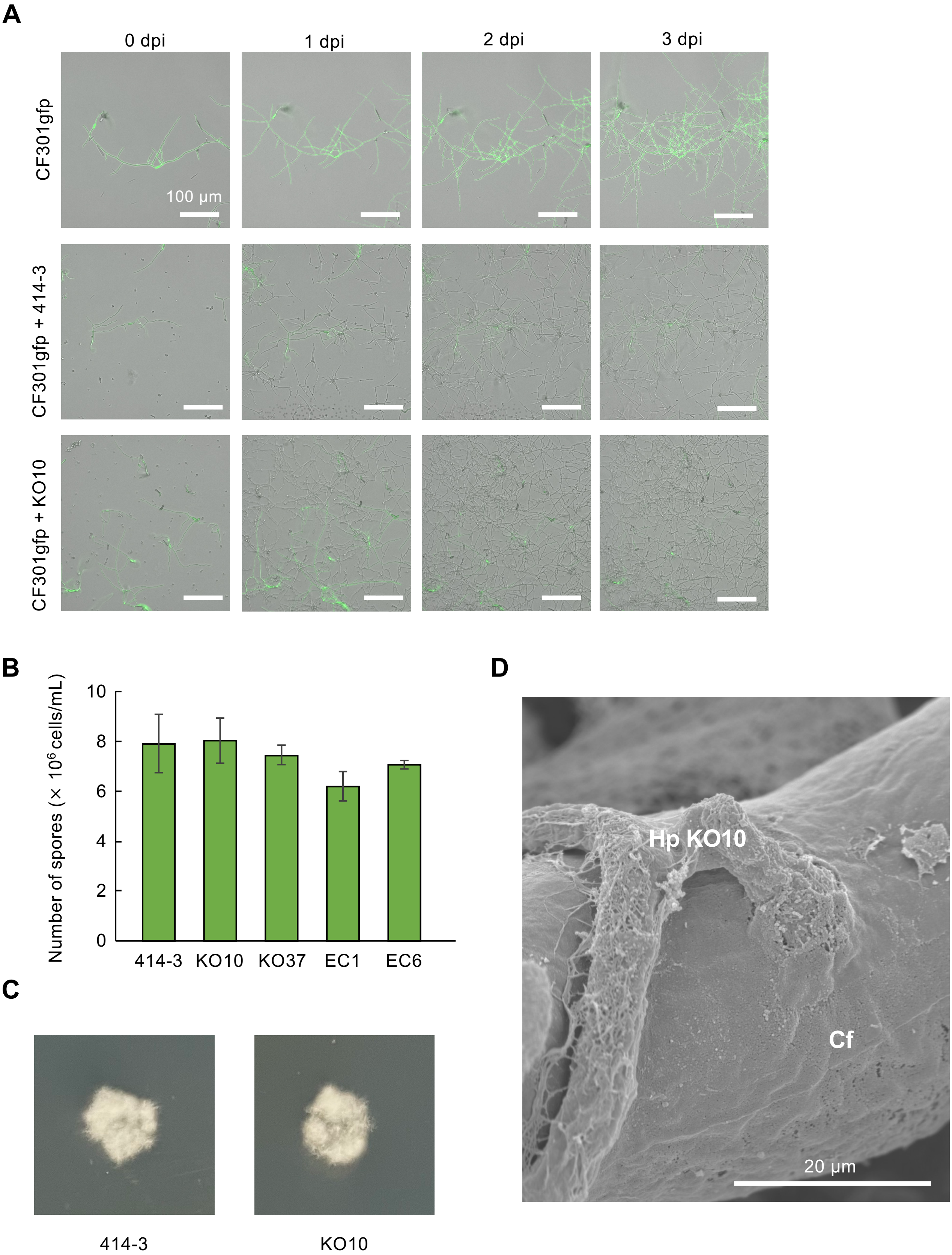

Fig. S9. ***In vitro*** **assay of** ***Hansfordia pulvinata*** **mycoparasitic activity against GFP-expressing** ***Cladosporium fulvum.*** (A) *H. pulvinata* wild-type 414-3 or Δ*HpDPH1* mutant strain KO10 were cocultured with *C. fulvum* CF301gfp, which constitutively expresses GFP, in MM broth without a carbon source at 25 ºC as described in Supplemental Methods. The parasitized *C. fulvum* cells lost GFP fluorescence. Bars indicate 100 μm. (B) Number of spores of 414-3, Δ*HpDPH1* mutants (KO10 and KO37) and ectopic strains (EC01 and EC06). Values are means of three biological replicates. Error bars indicate the standard deviation. (C) Mycelial growth of *H. pulvinata* 414-3 and KO10 strains on PDA.（D) Scanning electron micrographs of hyphae of *H. pulvinata* strain KO10 (Hp) parasitizing hyphae of *C. fulvum* (Cf). Bar indicates 20 μm.

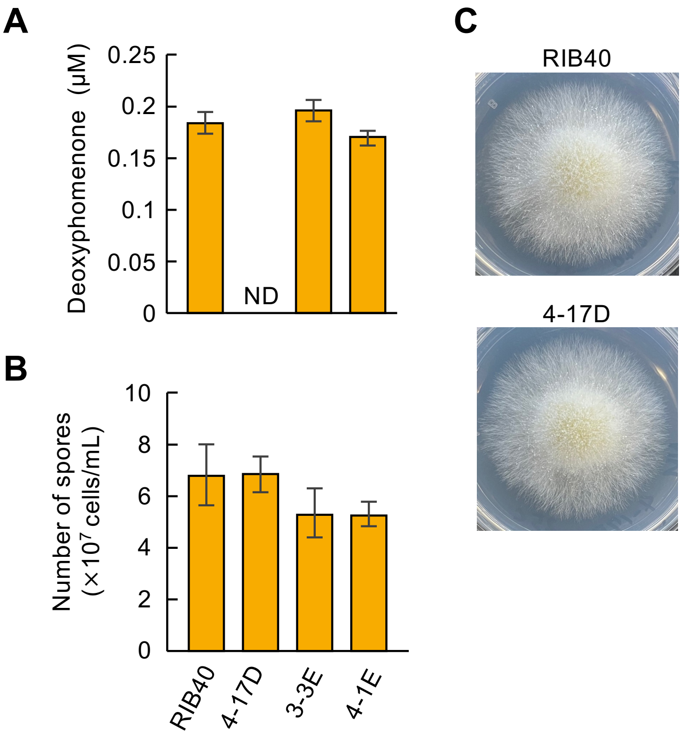

Fig. S10. Deoxyphomenone production and sporulation of *A. oryzae* RIB40 and transformants. Wild-type RIB40, Δ*AoDPH1* mutant 4-17D, and ectopic strains 3-3E and 4-1E were cultured in MM broth or agar. Values in A and B are means of three replicates. Error bars indicate the standard deviation. (**A**) LC-MS/MS quantification of deoxyphomenone in culture filtrates. ND, not detected. (**B**) Number of spores formed on agar. (**C**) Colony morphology of wild-type RIB40 and Δ*AoDPH1* mutant 4-17D on MM agar 25°C.

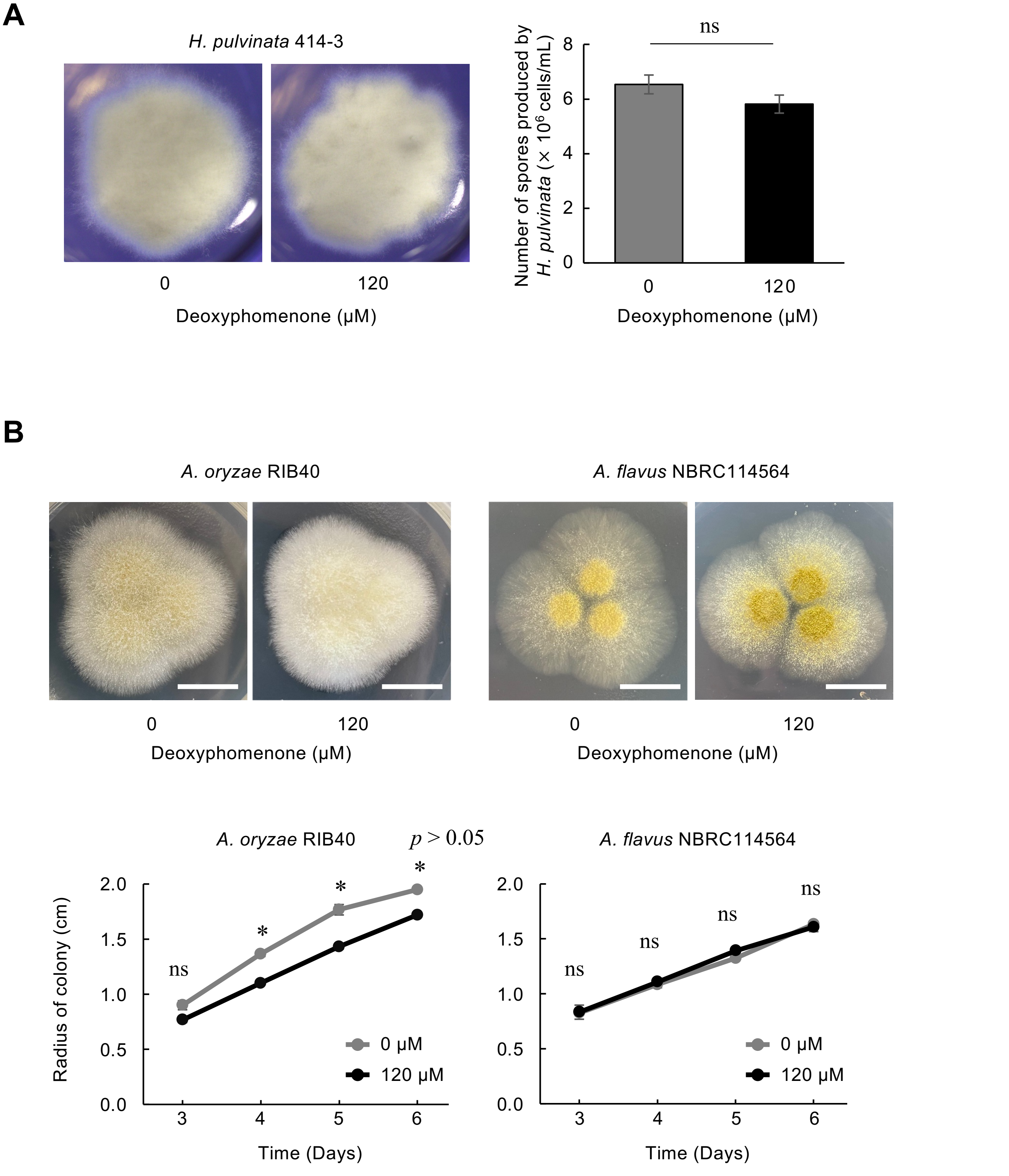

Fig. S11. Effect of deoxyphomenone on mycelial growth of *Hansfordia pulvinata, Aspergillus oryzae* and *A. flavus*. Strains were grown on MM agar with 1% methanol (0 μM) or 120 μM deoxyphomenone. Values are means of three biological replicates (± SD). Means were compared for significant differences amount treatments using Tukey's test. ns, no significance. (**A**) Colony morphology and number of spores produced by *H. pulvinata* 414-3. (**B**) Colony of *A. oryzae* RIB40 and *A. flavus* NBRC114564. Bars = 1 cm. Fewer ocherous spores were produced by RIB40 in the presence of deoxyphomenone but more were produced by NBRC114564.

Group	Species	*A. oryzae*	*A. flavus*	*A. parvisclerotigenus*	*A. minisclerotigenes*	*A. caelatus*	*A. pseudocaelatus*	*A. nakazawae*	*A. neoauricomus*	*A. ostianus*	*A. sesamicola*	*A. westerdijkiae*	*A. petrakii*	*H. pulvinata*
Group I (section *Flavi*)	*A. oryzae*	100.0												
	A. flavus	98.1	100.0											
	A. parvisclerotigenus	98.0	96.7	100.0										
	A. minisclerotigenes	95.1	94.4	95.1	100.0									
	A. caelatus	88.2	87.8	89.3	89.0	100.0								
	A. pseudocaelatus	88.1	87.7	89.3	88.9	99.6	100.0							
Group II (section *Circumdati* or Unsectioned)	*A. nakazawae*	61.0	61.0	62.0	61.7	61.0	60.9	100.0						
	A. neoauricomus	60.5	60.6	62.0	61.5	60.7	60.6	97.4	100.0					
	A. ostianus	59.2	59.2	60.6	60.0	59.2	59.0	93.1	94.1	100.0				
	A. sesamicola	58.9	58.9	59.9	59.7	59.0	58.9	95.4	95.0	90.2	100.0			
	A. westerdijkiae	61.3	61.3	62.2	61.9	61.0	61.0	95.2	94.3	91.1	92.2	100.0		
	A. petrakii	59.4	59.6	60.8	60.3	59.7	59.6	94.3	95.5	93.6	91.4	92.2	100.0	
	H. pulvinata	58.9	59.1	60.6	59.7	53.6	53.6	56.6	56.8	51.8	54.4	53.3	52.0	100.0

Column groups: Group I (section *Flavi*): *A. oryzae* – *A. pseudocaelatus*; Group II (section *Circumdati* or Unsectioned): *A. nakazawae* – *A. petrakii*.

Fig. S12. Pairwise similarity of concatenated sequences of DPH biosynthesis proteins.

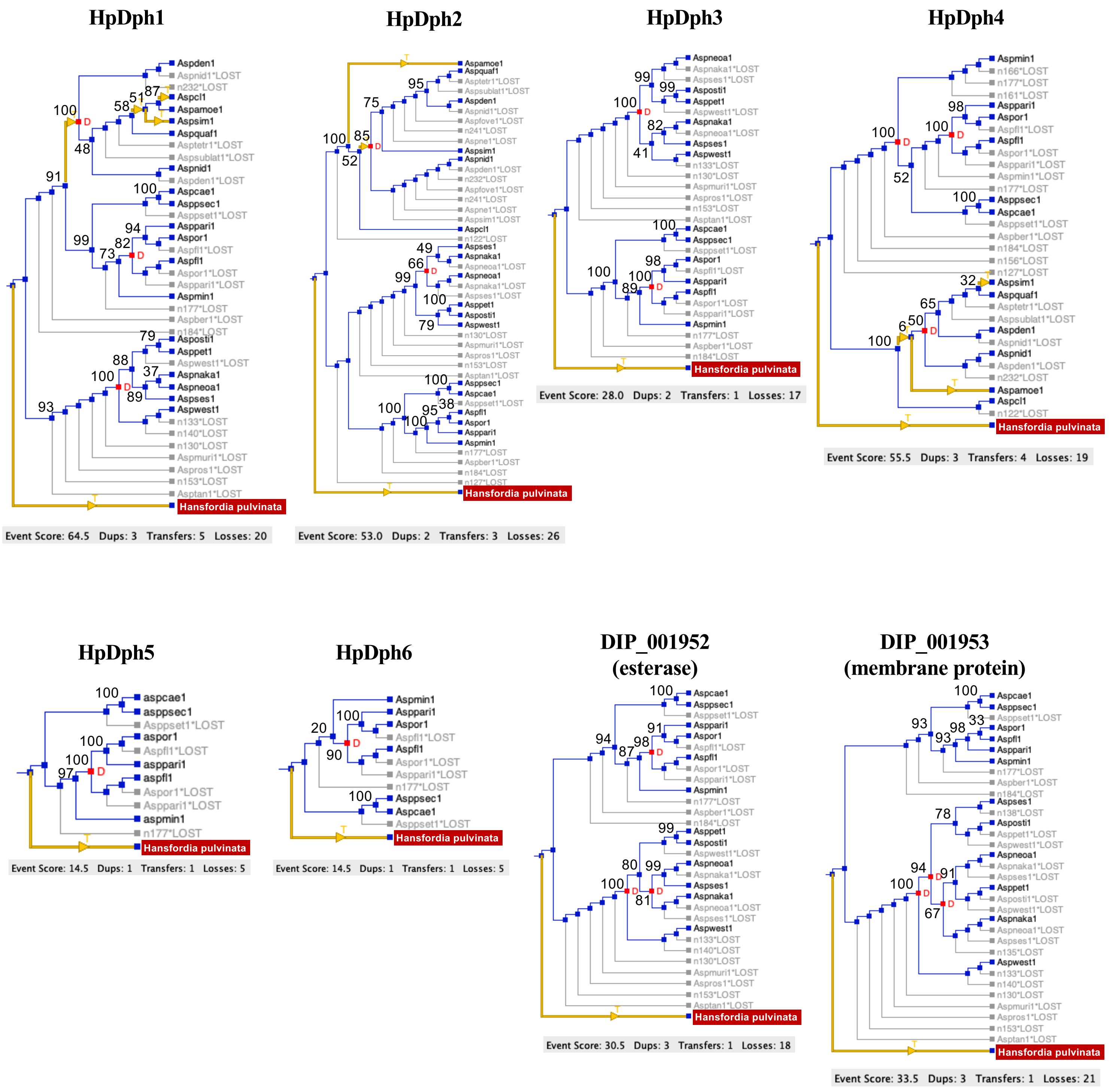

Fig. S13. Reconciliation of gene trees with species tree based on *DPH* nucleotide and amino acid sequences. Phylogenetic protein trees were compared with the species tree using NOTUNG v.2.9 to infer duplications, losses, and horizontal transfer events for the HpDph amino acid sequences. Numbers at branches represent bootstrap percentages (1000 replicates). Duplication nodes are marked by red squares with a red D; losses are in grey; migrations are indicated with yellow arrows. Horizontal gene transfer events for the HpDph1 (T1) and HpDph2/HpDph4 (T2) proteins are indicated by yellow stars. *Hansfordia pulvinata* is highlighted by a red box. Event scores were calculated as total cost of duplications, transfers and losses. Costs/weights were set as duplications (D), 1.5; transfers (T), 8.0; losses (L), 1.0 (ratio D:T:L is 1:5.3:0.67).

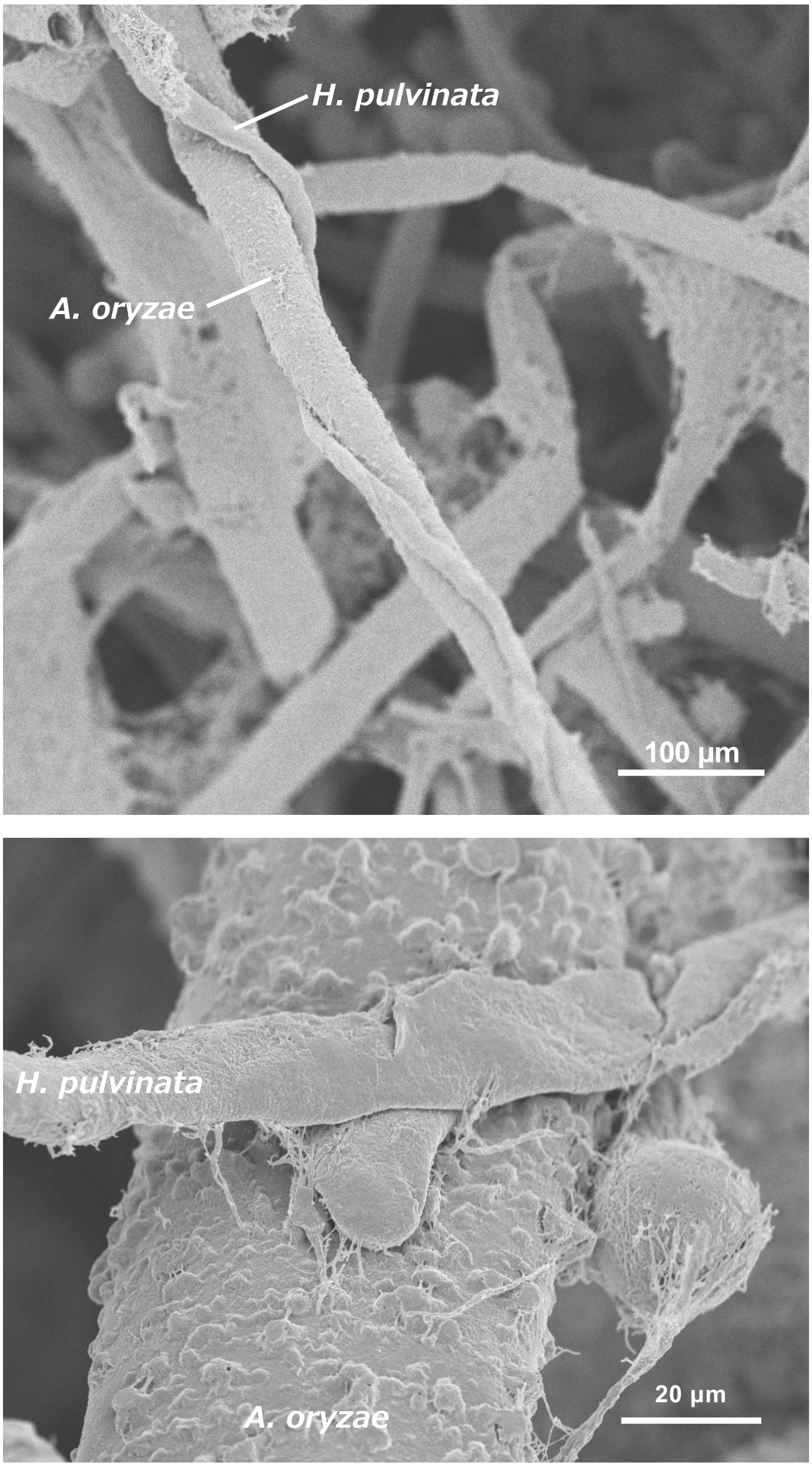

Fig. S14. **Hyphal contact between the mycoparasite *Hansfordia pulvinata* 414-3 and *Aspergillus oryzae* RIB40.**
Coiling of hyphae of 414-3 around the thick, rough hyphae of RIB40 was rarely found.
