# Supplementary material for: Adaptive evolution of sesquiterpene deoxyphomenone in mycoparasitism by Hansfordia pulvinata associated with horizontal gene transfer from Aspergillus species
Source: mBio. 2025 Mar 20;16(4):e04007-24. doi: 10.1128/mbio.04007-24 (PMC11980549; doi:10.1128/mbio.04007-24)
Supplement: Supplemental text — Supplemental methods. [file mbio.04007-24-s0002.docx]

**Supplemental Materials and Methods**

**Growth conditions for fungal strains and plants**

Strains of *H. pulvinata* 414-3 and *C. fulvum* CF301 were grown on PDA (half-strength, BD Difco, Franklin Lakes, NJ, USA), MM agar or broth (15 g sucrose, 5 g ammonium tartrate, 1 g NH_4_NO_3_, 1 g KH_2_PO_4_, 0.5 g MgSO_4_⋅7H_2_O, 0.1 g NaCl, 0.1 g CaCl_2_⋅H_2_O, 25 μL 0.2 mg/mL biotin, 15 g agar and 1 mL trace elements per liter) at 25 °C in the dark for 1 and 2 weeks, respectively, then spores were collected in sterile distilled water. *A. oryzae* and *A. flavus* strains from NITE-NBRC (www.nite.go.jp/nbrc/catalogue/) were cultured on PDA, MM agar and broth, and Czapek-Dox agar and broth (3 g NaNO_3_, 2 g KCl, 1 g KH_2_PO_4_, 0.5 g MgSO_4_⋅7H_2_O 20 g glucose and 15 g agar per liter adjusted to pH 6.5) at 30 °C in the dark for a week, and spores were collected in sterile distilled water containing 1% (v/v) Tween 20. The concentration of spores was determined using a hemocytometer. Strains were maintained on PDA at 25 °C for a few months. Spore suspensions containing 10% v/v glycerol of all fungi except *H. pulvinata* were preserved at –80 °C for long-term storage; *H. pulvinata* was stored in 10% v/v glycerol with 5% w/v trehalose.

For examining mycoparasitic interactions on tomato leaves, tomato cultivar Moneymaker, which lacks any apparent *Cf* resistance gene, was grown in plastic pots for 3 weeks in a climate chamber at 25 °C with 16 h light/8 h dark. Seedlings were transplanted to soil in the greenhouse and grown for 3 weeks. The lower sides of tomato leaves were sprayed with a spore suspension of *C. fulvum* CF301 (1 × 10^5^ spores/mL). After 2 weeks, a spore suspension of *H. pulvinata* 414-3 (1 × 10^5^ spores/mL) was sprayed on the brown lesions that had formed on the abaxial surfaces of the leaves. White mycelial patches of *H. pulvinata* were observed a week after inoculation.

**Electron microscopy**

*C. fulvum* CF301 or *A. oryzae* RIB40 were cultured on a nylon membrane (0.45 μm; GVS Japan, Tokyo, Japan) placed on PDA in a petri dish and incubated as described above. The membranes were then transferred to water agar and incubated for a week in the same conditions. A spore suspension of *H. pulvinata* (1 × 10^5^ spores/mL) was sprayed on the test colony on the membrane, then white colonies were observed 1 week later.

Small pieces (1 × 1 cm^2^) were cut from the membrane and fixed in 2.5% (v/v) glutaraldehyde in 100 mM cacodylate buffer (pH 7.4) overnight at 4 °C, then washed three times in the buffer, fixed in 1% (w/v) osmium tetroxide in the buffer for 1 h at room temperature and washed three times in distilled water. Specimens were then dehydrated through a graded ethanol series, critical-point dried in absolute ethanol using Leica EM CPD300 critical point dryer (Leica Microsystems, Wetzlar, Germany), then mounted on stubs, coated with platinum-palladium, and observed with a Hitachi SU-8220 scanning electron microscope (Tokyo, Japan).

For observing the ultrastructure of *C. fulvum* spores with transmission electron microscopy (TEM), spores of strain CF301 were treated with deoxyphomenone (120 µM) at 25 °C for 24 h in the dark. Spores were collected by centrifugation, washed three times with sterile water, then fixed in phosphate-buffered 2% glutaraldehyde at 4 °C overnight. The samples were again collected by centrifugation, then treated with 2% potassium permanganate in phosphate buffer for 1 h at room temperature and post-fixed in 2% w/v osmium tetroxide in phosphate buffer for 2 h at 4 °C. The samples were dehydrated in a graded ethanol series and embedded in epoxy resin (Fujifilm Wako, Osaka, Japan) at 60 °C for 48 hours. Ultrathin sections were cut using an ultramicrotome Leica UCT Ultracut (Leica Microsystems), then stained with uranyl acetate for 15 min and lead solution for 5 min and observed with a Hitachi TEM H-7600.

**Assays of antifungal activity and plant toxicity of deoxyphomenone**

*C. fulvum* spores were treated with deoxyphomenone [(+)-13-deoxyphomenone, sporogen-AO1; Apollo Scientific, Bredbury, UK] in 1% methanol (v/v) at 25 °C in the dark for 24 h. Then samples were observed for germination and hyphal elongation using a Nikon E600 light microscope (Tokyo, Japan).

The antifungal activity of deoxyphomenone was compared with that of an *N*-halo-alkylthioimide fungicide, captan (orthocide80; Arysta Life Science, Tokyo, Japan). *C. fulvum* spores (1 × 10^3^ cells/mL) were treated with distilled water, deoxyphomenone (120 µM), or captan (100 µM) on a hydrophobic TF0808 glass slide (Matsunami Glass, Osaka, Japan) for 24 h at 100% humidity, then each solution was replaced with fresh solution, and samples incubated another 24 h. Germinated spores were then counted using the Nikon light microscope. For the controls, dH_2_O or 1% methanol (v/v) were used. Each treatment was done in triplicate, and at least 200 spores and hypha were assessed for each treatment.

Spore suspensions of the strains *C. fulvum* CF301, *P. fuligena* Pf17923, kindly provided by K. Nakajima and T. Kawakami, and *F. oxysporum* f. sp. *lycopersici* CK3-1 were adjusted to 1 × 10^6^ spores/mL and 10 μL were dropped on MM agar supplemented with different concentrations of deoxyphomenone (20 to 120 μM). The strains were cultured at 25 °C in the dark for 2 weeks or 3 days.

To evaluate the toxicity of deoxyphomenone on tomato plants, we injected the abaxial surface of three leaves on each of two 1-month-old tomato plants with about 20 µL of deoxyphomenone (20 to 80 µM) or with 1% methanol (v/v) as a control. The plants were then incubated in a climate chamber for 1 week, then leaves were examined for necrosis.

**Phylogenetic analyses of deoxyphomenone biosynthetic gene clusters**

Deoxyphomenone biosynthetic genes and *DPH* clusters were initially identified in the genomic sequences of *H. pulvinata* 414-3 (Sushida et al., 2019) and *A. oryzae* RIB40 (Machida et al., 2005) using the BLAST program (blast.ncbi.nlm.nih.gov). Sequences shown in **Table S1 and S2** were aligned using MAFFT online version 7 (Katoh and Standley, 2013) with default parameters and trimmed using TrimAl version 1.4 (Capella-Gutiérrez et al., 2009). Maximum likelihood phylogenetic trees were constructed using RAxML version 8.2.12 with 1000 bootstraps using randum seed numbers. To compare the consistency of topology among the phylogenetic trees, we employed the following the Kishino-Hasegawa (KH) (Kishino and Hasegawa, 1989), the Shimodaira and Hasegawa (SH) (Shimodaira and Hasegawa, 1999), expected likelihood weights (ELW) (Strimmer and Rambaut, 2002), and approximately unbiased (AU) tests (Shimodaira, [2002](https://pmc.ncbi.nlm.nih.gov/articles/PMC9731855/#ece39553-bib-0059)) with 10,000 bootstrap replicates using the IQ‐TREE program version 1.6.12 (Trifinopoulos et al. 2016). Most appropriate phylogenetic trees were seleceted.

The PROTCATAUTO and GTRCAT models were selected for amino acid and nucleotide sequences, respectively (Stamatakis, 2014). Dot plot alignments were generated from the MAFFT analysis. Pairwise sequence alignments of *DPH6* homologous genes were performed using EMBOSS Needle (Madeira et al. 2022).

The phylogenetic species tree was constructed using maximum likelihood based on the 500 monocore genes (a single homolog in each of the species) using CVtree version 3.0 (*k* = 16) (Qi et al., 2004) by uploading the genomic information for the 60 published fungi shown in **Table S1**.

Reconciliation analysis between species and gene trees was performed using NOTUNG v.2.9 (Stolzer et al., 2012) according to the instructions (version 2.8 beta) to infer the evolutionary trajectories of the *DPH* clusters. Event scores were calculated as the total costs of duplications, transfers and losses. Costs/weights were set as duplications (D), 1.5; transfers (T), 8.0; losses (L), 1.0 (ratio D:T:L is 1:5.3:0.67) for amino acid sequences, and D, 1.5; T, 6.0; L, 1.0 (ratio D:T:L is 1:4:0.67) for nucleotide sequences for the gene clusters.

**Quantitative detection of deoxyphomenone**

*H. pulvinata* (1 × 10^6^ spores) was cultured in 50 mL of MM broth with shaking at 25 °C in a light/dark cycle of 16 h/8 h for 2 weeks. *A. oryzae* and *A. flavus* (1 × 10^6^ spores) were incubated in MM broth without shaking at 25 °C in the dark for 2 weeks. The culture supernatants were collected by centrifugation and filtered through a 0.45 μm filter (Merk, Darmstadt, Germany). Deoxyphomenone was quantified using a 4000 QTRAP LC-MS/MS system (Sciex, MA, US) equipped with a 1290 series HPLC system (Agilent Technologies, CA, US) as previously reported (Iida et al., 2018). Commercial deoxyphomenone (Apollo Scientific) was used as a standard.

**Construction of transformation vectors**

Genomic DNA was extracted from *H. pulvinata* and *A. oryzae* mycelia grown in MM broth for a week using Nucleo-Mag Plant (TakaraBio, Shiga, Japan) according to the manufacturer’s instructions. The DNA was then amplified by PCR using PrimeSTAR GXL Premix (TakaraBio) or KOD ONE (TOYOBO, Osaka, Japan) according to the instructions.

For generating the gene knockout vector, the upstream (1.2 kb) and downstream (1.0 kb) regions of *HpDPH1* were amplified from *H. pulvinata* genomic DNA using primer sets Dpprx2LF1/Dpprx2LR1 and Dpprx2UF1/Dpprx2UR1, respectively (**Table S3**). Geneticin-resistance gene (*gen*) cassettes were amplified from pRM254 (Mehrabi et al., 2015) using primers pRM254 attL1_F/pRM254 attL1_R. These primers contain a 5′ overhang sequence to overlap the sequences at the ends of the linearized plasmid or the *gen* cassette (**Table S3**). Plasmid pPM43GW was amplified using the primers pPM43GW_RB_F and pPM43GW_RB_R to linearize and remove the *ccdB* gene, which encodes a product that is lethal to bacterial cells. Four fragments were combined to generate the gene replacement vector pGW43_dpprx2ko using the In-Fusion EcoDry Cloning Kit (TakaraBio).

To introduce the *AoDPH1* gene into Δ*HpDPH1* strain KO10, *AoDPH1* (without introns) and hygromycin resistance gene (*hph*) cassettes were synthesized by VectorBuilder, Inc. (Chicago, IL, USA). The *AoDPH1* and *hph* genes were driven under the promoters of the *A. nidulans trpC* gene and *Cochliobolus heterostrophus* *gpd1* (NCBI accession X02390.1 and X63516.1), respectively. Synthesized DNA was inserted into the blunt end of the PvuII-digested plasmid pPZP-PvuII kindly provided by Prof. Chihiro Tanaka.

To construct gene replacement vector for *AoDPH1* and *AfDPH1* gene, a plasmid pSH75 (Kimura and Tsuge 1993) was digested with *Bgl* II and *Hin*d III, and the resulting 2.4 kb fragment was used as a backbone. Marker gene cassettes (4.7 kb) containing a pyrithiamine resistant gene (*ptrA*) and an *EGFP* gene cassette were amplified from a plasmid pPTREXeGFP using the primers pPTREXeGFP_F and pPTREXeGFP_R. The upstream (1.5 kb) and downstream (1.5 kb) regions of *AoDPH1* were amplified from genomic DNA of *A. oryzae* RIB40 using primer sets AoDPH1_LB_F/AoDPH1_LB_R and AoDPH1_RB_F/AoDPH1_RB_R, respectively (**Table S3**). These primers contain a 5′ overhang sequence to overlap the sequences at the ends of the pSH75 fragment or the marker cassette (**Table S3**). These four fragments were combined using the In-Fusion EcoDry Cloning Kit (TakaraBio), then the resulting plasmid was named pSH75PTRdAoDPH1. The plasmid was linearized by digestion with *Swa* I and used for fungal transformation.

Plasmid vectors were introduced into *Escherichia coli* DH5α (NIPPON GENE, Tokyo, Japan) and extracted using MagExtractor -Plasmid- (TOYOBO) according to the instructions. The correct orientation of the fragments in the final constructs was confirmed by PCR.

**Fungal transformation**

All transformations were done as previously described (Okmen et al., 2013) with some modifications for *H. pulvinata*. Briefly, *A. tumefaciens* (*Rhizobium radiobacter*) strain AGL-1 was transformed with the plasmid vectors used for the *H. pulvinata* transformation using the Gene Pulser electroporator (Bio-Rad Laboratories, Hercules, CA, USA) according to the instructions, then grown on LBman agar (10 g tryptone, 10 g NaCl, 5 g yeast extract, 10 g mannitol, 20 g agar per liter) supplemented with appropriate antibiotic at 28 °C for 3 days. Colonies were then collected and resuspended in IM broth (1 g glucose, 2.05 g K_2_HPO_4_, 1.45 g KH_2_PO_4_, 0.15 g NaCl, 0.50 g MgSO_4_·7H_2_O, 0.07 g CaCl_2_·2H_2_O, 0.5 g (NH_4_)_2_SO_4_, 0.5% (w/v) glycerol, 8.53 g 2-(*N*-morpholino)ethanesulfonic acid [pH 5.3] per liter) supplemented with 200 µM acetosyringone at an optical density at 600 nm (OD_600_) of 0.2. The bacterial suspension (200 µL) was mixed with 200 µL of a spore suspension of *H. pulvinata* (1.0 × 10^5^ cells/mL), then the suspension was spread on a nylon membrane (GVS Japan) that had been placed on IM agar (IM; 20 g per liter) in a plate. After 2 days of incubation in the dark at 25 °C, the membranes were transferred to PDA supplemented with either 200 µg/mL geneticin (G418 sulfate; Fujifilm Wako) or 100 µg/mL hygromycin (Fujifilm Wako), and 50 µg/mL meropenem trihydrate (Fujifilm Wako). Transformed fungal colonies appeared after a week were transferred to new PDA plates supplemented with geneticin (Fujifilm Wako) or hygromycin (Fujifilm Wako) and meropenem trihydrate (Fujifilm Wako) at the same concentration as described above and incubated in the dark at 25 °C for a week. Gene replacement was confirmed by PCR amplification using the primers listed in **Table S3**.

Protoplasts of *A. oryzae* RIB40 and *A. flavus* NBRC114564 were transformed using polyethylene glycol. First, 2.4 × 10^8^ spores harvested from a 10-d-old culture on Czapek-Dox agar were resuspended in 200 mL of Czapek-Dox broth and incubated at 30 °C at 160 rpm for 18 h. The mycelium was then collected using miracloth (Merck, NJ, USA) and washed once with distilled water, then incubated in 20 mL of a protoplast solution consisted of 10 mM phosphate buffer (pH6.0), 0.8 M NaCl, 10 mg/mL Lysing enzyme (Sigma-Aldrich, MO, USA), 5 mg/mL Cellulase Onozuka R-10 (Yakult Pharmaceutical, Tokyo, Japan), and 2.5 mg/mL Yatalase (Takara, Shiga, Japan) at 30 °C with shaking at 83 rpm for 3 h. The protoplasts were filtered through a mesh and centrifuged at 2000 × *g* and 4 °C for 5 min. The pellet was washed once in 0.8 M NaCl and centrifuged at 2000 × *g* and 20 °C for 5 min, then resuspended in 1.2 mL of solution 1 (Sol1: 9.35 g NaCl, 2 mL 1 M CaCl_2_, 2 mL 1 M Tris-HCl per 200 mL). Two handred fourty microliters of solution 2 (Sol2: 40% (w/v) PEG4000, 10 mL 1 M CaCl_2_, 10 mL 1 M Tris-HCl per 200 mL) was added to the protoplast suspension and the resulting mixture was dispensed 300 μL each into 14-mL round tubes. A linearized vector DNA was added to the mixture and held on ice for 30 min, then 1 mL of Sol2 was added and held at 25 °C for 20 min. The protoplasts were washed once in 10 mL of Sol1, and centrifuged at 2000 × *g* at 20 °C for 5 min. After the supernatant was discarded, the protoplasts were resuspended in 300 μL of Sol1, then placed on a regeneration selection medium consisted of Czapek-Dox agar supplemented with 5% (w/v) NaCl, 0.1 μg/mL pyrithiamine hydrobromide (Sigma-Aldrich, MO, USA). The resulting plates were overlayed with 5 mL of soft agar medium consisted of 5% (w/v) NaCl and 0.5% (w/v) agar, and incubated at 30 °C for 4 d. The transformants grown on the regeneration selection medium were isolated and transferred to Czapek-Dox agar containing 0.2 μg/mL pyrithiamine hydrobromide and incubated at 30 °C for another selection. Gene replacement of the transformants was confirmed by PCR amplification using the primers listed in **Table S3**.

**Quantitative real-time PCR**

*C. fulvum* CF301 and *H. pulvinata* 414-3 were cultured on nylon membranes (GVS Japan) on PDA at 25 °C in the dark for 1 and 2 weeks. The membranes were transferred to water agar and then incubated in the same conditions for a week. *C. fulvum* colonies were sprayed with a spore suspension of *H. pulvinata* (1 × 10^5^ spores/mL) and checked for white colony growth of *H. pulvinata* 1 week later. Total RNA was extracted from the mycelia using the RNeasy Plant Mini Kit (Qiagen, Hilden, Germany). cDNA libraries were generated using SuperScript IV VILO Master Mix with ezDNase Enzyme (ThermoFisher Scientific, Waltham, MA, USA) according to the manufacturer’s instructions. Primers for quantitative real-time PCR of 90–150-bp fragments of cDNA were designed (**Table S3**), and the PCR was run on the LightCycler 480 System (Roche, Basel, Switzerland) using the KAPA SYBR Fast qPCR Kit (Nippon Genetics, Tokyo, Japan) according to the manufacturer’s instructions. Relative expression levels were calculated using the comparative CT (2^−∆∆CT^) method (Livak and Schmittgen, 2001). The data were normalized to the transcript level of the *H. pulvinata* actin gene (**Table S3**). Transcript levels of target genes in each RNA sample were measured for three independent experiments, each with two replicates.

**Statistical analyses**

Means and standard deviations of number of spores, spore germination, and hyphal length were calculated. Significant differences (*P* < 0.05) in the number of spores produced by the different fungal strains, percentage spore germination and hyphal lengths of *C. fulvum*, and colony diameter of *Aspergillus* strains were evaluated using either Tukey’s test or Williams’ multiple comparison. Significant differences (*P* < 0.05) in gene expression levels were determined using Welch’s *t*-test followed by Bonferroni–Holm correction for multiple testing. All data were analyzed in the program R version 4.0.3 ([www.r-project.org](http://www.r-project.org)).

**In-well assay and observation of mycoparasitism**

Green fluorescence protein (GFP)-expressing *C. fulvum* strain CF301gfp (Iida et al., 2018) and *H. pulvinata* strains were cultured on PDA as mentioned above to prepare spore suspensions of CF301gfp (5 × 10^4^ spores/mL) and *H. pulvinata* (1 × 10^6^ spores/mL). Then 50 µL of a spore suspension of CF301gfp and of each *H. pulvinata* strain were added to 100 µL of MM broth without a carbon source (MM without sucrose) in each well of a 48-well plate. At least 8 wells were used per treatment, and CF301gfp was used as the control. The plates were incubated at 25 °C in the dark for 3 d. Hyphae were observed using a fluorescence microscope BZ-X800 (KEYENCE, Osaka, Japan); lack of GFP fluorescence from *C. fulvum* was correlated with the mycoparasitic activity of *H. pulvinata* using the hybrid cell count system of the microscope. We named this simple method for detecting mycoparasitism the IWAO method, derived from “in-well assay and observation” and in honor of the method’s developer, E. Iwao.

**REFERENCES**

Capella-Gutiérrez, S., Silla-Martínez, J.M., and Gabaldón, T. (2009) trimAl: a tool for automated alignment trimming in large-scale phylogenetic analyses. *Bioinformatics* 25: 1972-1973.

Iida, Y., Ikeda, K., Sakai, H., Nakagawa, H., Nishi, O., and Higashi, Y. (2018) Evaluation of the potential biocontrol activity of *Dicyma pulvinata* against *Cladosporium fulvum*, the causal agent of tomato leaf mold. *Plant Pathology* 67: 1883-1890.

Katoh, K., and Standley, D.M. (2013) MAFFT multiple sequence alignment software version 7: improvements in performance and usability. *Molecular Biology and Evolution* 30: 772-780.

Kimura, N., and Tsuge, T. (1993) Gene cluster involved in melanin biosynthesis of the filamentous fungus *Alternaria alternata*. *Journal of bacteriology* 175: 4427-4435.

Kishino, H., and Hasegawa, M. (1989) Evaluation of the maximum likelihood estimate of the evolutionary tree topologies from DNA sequence data, and the branching order in hominoidea *Journal of Molecular Evolution* 29:170-179.

Ökmen, B., Etalo, D. W., Joosten, M. H. A. J., Bouwmeester, H. J., de Vos, R. C. H., Collemare, J., & de Wit, P. J. G. M. (2013) Detoxification of alpha-tomatine by *Cladosporium fulvum* is required for full virulence on tomato. *The New phytologist* 198: 1203-1214.

Livak, K.J., and Schmittgen, T.D. (2001) Analysis of Relative Gene Expression Data Using Real-Time Quantitative PCR and the 2−ΔΔCT Method. *Methods* 25: 402-408.

Machida, M., Asai, K., Sano, M., Tanaka, T., Kumagai, T., Terai, G. et al. (2005) Genome sequencing and analysis of *Aspergillus oryzae*. *Nature* 438: 1157-1161.

Madeira, F., Pearce, M., Tivey, A.R.N., Basutkar, P., Lee, J., Edbali, O., Madhusoodanan, N., Kolesnikov, A., and Lopez, R. (2022) Search and sequence analysis tools services from EMBL-EBI in 2022. *Nucleic Acids Research* 50(W1): W276-W279.

Mehrabi, R., Mirzadi Gohari, A., da Silva, G.F., Steinberg, G., Kema, G.H.J., and de Wit, P.J.G.M. (2015) Flexible gateway constructs for functional analyses of genes in plant pathogenic fungi. *Fungal Genetics and Biology* 79: 186-192.

Qi, J., Luo, H., and Hao, B. (2004) CVTree: a phylogenetic tree reconstruction tool based on whole genomes. *Nucleic Acids Research* 32: W45-W47.

Stamatakis, A. (2014) RAxML version 8: a tool for phylogenetic analysis and post-analysis of large phylogenies. *Bioinformatics* 30: 1312-1313.

Stolzer, M., Lai, H., Xu, M., Sathaye, D., Vernot, B., and Durand, D. (2012) Inferring duplications, losses, transfers and incomplete lineage sorting with nonbinary species trees. *Bioinformatics* 28: i409-i415.

Shimodaira, H., and Hasegawa, M. (1999) Multiple comparisons of log-likelihoods with applications to phylogenetic inference *Molecular Biology and Evolution* 16:1114-1116.

Shimodaira H. (2002) An approximately unbiased test of phylogenetic tree selection I *Systematic Biology* 51:492-508.

Strimmer, K., and Rambaut, A., (2002) Inferring confidence sets of possibly misspecified gene trees *Proceedings of the Royal Society B: Biological Sciences* 269:137-142.

Sushida, H., Sumita, T., Higashi, Y., and Iida, Y. (2019) Draft genome sequence of *Dicyma pulvinata* Strain 414-3, a mycoparasite of *Cladosporium fulvum*, causal agent of tomato leaf mold. *Microbiology Resource Announcements* 8.

Trifinopoulos, J., Nguyen, L.T., von Haeseler, A., Minh, B.Q. (2016) W-IQ-TREE: a fast online phylogenetic tool for maximum likelihood analysis. *Nucleic Acids Research* 44: W232-235.
